# Crop rotation patterns affect the growth, soil properties, and rhizosphere microbiome of cut chrysanthemums

**DOI:** 10.3389/fmicb.2026.1763144

**Published:** 2026-05-28

**Authors:** Chaoyue Tang, Huayang Li, Xiong Shi, Hong Ge, Yaping Kou, Shuhua Yang, Ruidong Jia, Xin Zhao

**Affiliations:** 1 State Key Laboratory of Vegetable Biobreeding, Key Laboratory of Biology and Genetic Improvement of Flower Crops (North China), Ministry of Agriculture and Rural Affairs, Institute of Vegetables and Flowers, Chinese Academy of Agricultural Sciences, Beijing, China; 2 National Nanfan Research Institute of CAAS, Sanya, China

**Keywords:** chrysanthemum, continuous cropping obstacles, crop rotation, rhizosphere microorganisms, soil properties

## Abstract

**Background:**

Continuous cropping obstacles in cut chrysanthemum, which are characterized by soil nutrient imbalance, reduced enzyme activities, and disrupted rhizosphere microbial communities, restrict the development of its industry. This study investigated the regulatory effects of crop rotation on soil properties and microbial communities, and compared the mitigation efficiency of different rotation patterns.

**Results:**

At 60 days of growth, cut chrysanthemums under crop rotation systems exhibited significant increases in stem diameter, as well as fresh and dry weights of both aboveground and underground biomass, compared to continuous cropping. Rotation significantly increased soil total nitrogen, hydrolyzable nitrogen, and available phosphorus, with cabbage rotation exhibiting the most prominent phosphorus accumulation effect. The activities of soil catalase, alkaline phosphatase, and sucrase were higher in rotation groups, whereas the activity of urease decreased with successive planting cycles. Bacterial richness increased with planting cycles, while fungal diversity declined. Notably, rotation reduced the relative abundance of pathogenic *Fusarium* by 17.1–28.1%. Multivariate analyses indicated that soil nitrogen and phosphorus were closely correlated with bacterial community structure, while phosphorus was the most influential factor on fungal communities. Critically, the two crop rotation systems exhibited distinct mechanisms: maize primarily exerts regulatory effects on soil microbial community structure and enzyme activities, while cabbage focuses on optimizing soil nutrient element status.

**Conclusion:**

Crop rotation with maize or cabbage alleviates continuous cropping obstacles by improving soil nutrient status, enhancing enzyme activities, and optimizing rhizosphere microbial communities. Maize rotation excels in regulating soil enzyme activities and bacterial communities, whereas cabbage rotation is more effective in promoting plant biomass during the vegetative growth stage, accumulating soil phosphorus, and inhibiting pathogenic fungi. This study provides a theoretical basis for sustainable cut chrysanthemum production via rotation management strategies designed to enhance soil microbial and physicochemical properties.

## Introduction

*Chrysanthemum morifolium*, renowned as one of China’s “Top Ten Famous Flowers” and among the four major cut flowers globally, enjoys widespread popularity and occupies a pivotal position in the floriculture industry. In China, the cut chrysanthemum industry has experienced consistent annual growth; its cultivation area exceeds 7,000 hectares, with annual sales revenue surpassing 1.5 billion yuan (Wang et al., 2022). However, a major challenge in its production is the continuous cropping obstacle, which is recognized as a key factor limiting yield and quality (Li et al., 2018). These obstacles not only diminish the economic returns of enterprises and farmers but also significantly hinder the overall development of the cut chrysanthemum industry. The problems associated with continuous cropping include elevated soil pH, nutrient imbalance, disruption of microbial community structure, and soil compaction (Liu et al., 2025; Li C. et al., 2024; Yu et al., 2024), all of which exert severe adverse effects on the growth and development of *Chrysanthemum morifolium*.

Soil nutrients are fundamental to plant growth and development, as their balance and availability directly determine plant health and productivity (Wang K. et al., 2024). Nutrient deficiencies impose significant growth constraints and markedly reduce plants’ stress resistance against external factors such as drought, diseases, and pests (Xu et al., 2023). This issue is particularly prominent in the context of continuous crop monoculture. Under long-term continuous cropping, crops continuously and unilaterally absorb specific elements (e.g., nitrogen, phosphorus, and potassium) from the soil, disrupting the original soil nutrient equilibrium (Wang K. et al., 2024). Such imbalance not only directly impairs the normal growth of crops, resulting in reduced yield and deteriorated quality, but also causes economic losses in agricultural production, contradicting the principles of sustainable agriculture. This is particularly relevant for *Chrysanthemum morifolium*, a species with high nutritional demands. Studies have confirmed that its long-term monoculture leads to a significant decline in key soil nutrients, including nitrogen, phosphorus, and potassium (Liu et al., 2022), providing direct evidence of how continuous cropping disrupts soil nutrient balance.

Rhizosphere microorganisms are integral to plant growth and development, primarily through regulation of soil enzyme activity and soil nutrient transformation processes, such as nitrogen fixation, nitrification, and the decomposition and synthesis of humus (Bahram et al., 2018; Philippot et al., 2023). Specific microorganisms can enhance plants’ salt and alkali tolerance (Guo et al., 2025), activate plant peroxidase genes (Li et al., 2025), and improve the acquisition of key nutrients such as phosphorus (Sören et al., 2025). The application of beneficial microorganisms has been shown to increase plant immunity by up to 40%, thereby reducing losses by 5–20% (Yang et al., 2025). A stable microbial community composition is essential for maintaining a healthy farmland ecological environment; conversely, microbial dysbiosis can lead to the deterioration of soil nutrient status and enzymatic activity, ultimately compromising crop quality (Peng et al., 2025). Continuous cropping disrupts the stability of farmland soil microbial communities, decreases the biodiversity of rhizosphere soil bacteria (Li et al., 2023b), and increases the total number of pathogenic fungi (e.g., *Fusarium oxysporum*; Wang et al., 2017; Miao et al., 2023). This imbalance can induce *Fusarium* wilt in chrysanthemums (Pheirim and Sobita, 2024), posing a significant threat to the growth of *Chrysanthemum morifolium*.

Common strategies to address continuous cropping obstacles include modifying cropping systems, augmenting organic fertilizer application, and using soil amendments (Zheng et al., 2024; Chen et al., 2022; Andrade et al., 2023). Among these, crop rotation stands out as a widely adopted, environmentally sustainable cropping system proven effective in preventing such obstacles (Yang et al., 2023; Liu et al., 2024; Yang et al., 2024). Diverse rotation patterns in agriculture—such as soybean-maize rotation (Zhao et al., 2024; Luo et al., 2024), highland barley-wheat rotation (Wu et al., 2025), and soybean-rape rotation have successfully balanced soil nutrient utilization (Fatima et al., 2025), suppressed the diseases and pests incidence, regulated soil fertility, and improved soil microbial community composition. However, research on crop rotation in chrysanthemums, particularly in cut chrysanthemums, remains limited, highlighting a significant gap and opportunity for its application in this high-value sector.

The continuous production system for cut chrysanthemums induces severe continuous cropping obstacles, reducing economic benefits. To identify effective mitigation strategies, this study introduced crop rotation systems, pairing cut chrysanthemum with either *Zea mays* L. or *Brassica oleracea*. Comparative analyses were conducted on indicators such as plant phenotypic and physiological traits, soil physicochemical properties, and rhizosphere microorganisms between these rotation groups and the group with continuous cropping of cut chrysanthemums. The objective was to identify the key factors impeding chrysanthemum growth and soil health under continuous cropping, thereby providing a scientific basis for developing sustainable production models for the cut chrysanthemum industry.

## Experimental site, materials, and methods

### Experimental site, growing conditions and field experimental design

This experiment was conducted at the production base of Jiazhihui Agricultural Co., Ltd. in Dongfang City, Hainan Province (18°43′N, 108°36′E). The site has a typical tropical monsoon marine climate, with small annual temperature differences, sufficient annual sunshine, and abundant precipitation, which is highly suitable for year-round continuous production of cut chrysanthemums. Prior to the initiation of this experiment, the field had been under intensive continuous monoculture of cut chrysanthemums for more than 5 years, with uniform soil physicochemical properties and consistent continuous cropping history across the experimental area, providing a representative and homogeneous test platform for this study.

The experiment encompassed three consecutive cropping cycles from September 2023 through January 2025. The first (September 2023–January 2024) and third (September 2024–January 2025) cycles were both planted with cut chrysanthemum across all treatments, followed the enterprise’s standard cultivation procedures. The second cycle (January–May 2024) served as the intervention period, during which the following three treatments were applied: (1) continuous cropping (T-JH), with a second cycle of cut chrysanthemum; (2) maize rotation (T-YM), fresh maize (*Zea mays*) grown for fresh ears, following a full growth season; and (3) cabbage rotation (T-GL), with a full-season crop of cabbage (*Brassica oleracea*). For the cabbage rotation, seeds were sown in February 2024, followed by a 30-day nursery period, and seedlings were transplanted in March and harvested in May 2024.

The experiment was arranged in a Randomized Complete Block Design (RCBD) with three treatments and three independent biological replicates per treatment, each serving as an independent block, resulting in a total of nine experimental plots. Each plot was 21 m^2^ in size, with a 30 cm wide isolation belt between adjacent plots. All agronomic management practices (including irrigation, fertilization, pruning, and pest control) were applied uniformly across all plots during the experiment, following the enterprise’s standard cultivation procedures in the region, with no additional fertilization or pesticide application during the second crop of rotation crops.

### Plant materials and phenotypic measurements

The plant varieties used in the experiment were as follows: cut chrysanthemum (*Chrysanthemum morifolium* Ramat. cv. ‘Guangyu’), maize (*Zea mays* L. cv. ‘Hongtaiyang’), and cabbage (*Brassica oleracea* var. *capitata* L. cv. ‘Zhonggan 628’). Cut chrysanthemum plant samples were collected at 30, 60, and 90 days after planting during each cropping cycle with three biological replicates of each time point. The measured phenotypic traits included plant height, stem diameter, fresh stem weight, fresh root weight, dry stem weight, and dry root weight.

### Plant and soil sampling methods

For the three crops of cut chrysanthemum, plant samples were collected at 30, 60, and 90 days after planting, corresponding to the early vegetative growth stage, early reproductive growth stage, and commercial harvest stage, respectively. Three independent biological replicates were set for each treatment at each sampling time point, with 3 uniform plants collected per replicate. We systematically analyzed the dynamic changes in six key phenotypic indices of cut chrysanthemums under different planting treatments, which were all clearly defined and measured in the Methodology section, including: plant height, stem diameter, fresh stem weight, fresh root weight, dry stem weight, and dry root weight.

Soil was sampled at 30, 60, and 90 days after planting and physicochemical analyses were done as follows: For bulk soil sampling, soil surrounding the roots at a depth of approximately 10–15 cm from the ground surface was collected after uprooting the plants. The soil from around the roots of every three plants was combined to form one composite sample. Rhizosphere soil refers to the soil adhering to the root surface within 1–2 mm. After cutting the roots, they were placed in a 50 mL centrifuge tube and eluted with 0.2 × PBS eluent. After removing the roots, the solution was centrifuged at 4000 r/min for 10 min, and the supernatant was discarded. The rhizosphere soil from every 3 plants was pooled to form one composite sample.

### Cut chrysanthemum phenotypic measurement and soil physicochemical analysis

The six phenotypic indices measured in this study are defined as follows:

Plant height: Natural height from the stem base to the apical growing point, measured with a measuring tape; Stem diameter: Stem diameter at 2 cm above the soil surface, measured with a digital vernier caliper; Fresh stem weight: Fresh weight of the aboveground stem (core commercial organ of cut chrysanthemum), weighed immediately after rinsing and blotting surface water; Fresh root weight: Fresh weight of the intact underground root system, weighed immediately after rinsing and blotting surface water; Dry stem/root weight: Constant weight after oven-drying at 105 °C for 30 min followed by 75 °C to constant weight. All soil analyses were performed according to the 3rd edition of Soil Agrochemical Analysis, 2000 (Bao, 2000). Eleven soil indicators were systematically quantified, including:

Soil pH: Determined potentiometrically using a precision benchtop pH meter (FE28, Mettler Toledo, Switzerland) with a soil-to-deionized water ratio of 1:2.5 (w/v). The soil suspension was thoroughly stirred for 5 min and stood for 30 min before measurement, with 3 technical replicates set for each sample.

Soil organic matter (SOM) and organic carbon (SOC): SOM content was determined via the potassium dichromate oxidation-external heating method. Briefly, 0.2 g of air-dried soil sample was digested with 0.8 mol/L K₂Cr₂O₇-H₂SO₄ solution in a boiling water bath at 170–180 °C for 5 min, and the residual K₂Cr₂O₇ was titrated with 0.2 mol/L FeSO₄ standard solution. SOC content was converted from SOM content using the standard conversion coefficient of 1.724 (SOC = SOM / 1.724).

Total nitrogen (TN): Determined via the concentrated H₂SO₄ digestion-Kjeldahl method. 0.5 g of air-dried soil sample was digested with concentrated H₂SO₄ and catalyst mixture at 380 °C for 2 h, and the digested solution was quantified using an automatic Kjeldahl nitrogen analyzer (K9840, Hanon, China).

Hydrolyzable nitrogen (HN): Quantified by the alkaline hydrolysis-diffusion method. 1.0 g of air-dried soil sample was placed in the outer chamber of a diffusion dish, with 2% H₃BO₃ indicator solution added to the inner chamber. 10 mL of 1.8 mol/L NaOH solution was quickly added to the outer chamber, and the dish was immediately sealed and incubated at 40 °C for 24 h. The H₃BO₃ solution was then titrated with 0.01 mol/L HCl standard solution to calculate HN content.

Total phosphorus (TP): Determined by the NaOH fusion-molybdenum antimony anti-colorimetric method. 0.25 g of air-dried soil sample was fused with NaOH in a nickel crucible at 720 °C for 15 min, and the melt was dissolved with deionized water and filtered. The filtrate was developed with molybdenum antimony anti-reagent, and TP content was determined colorimetrically at 700 nm using a UV–visible spectrophotometer (UV-1800, Shimadzu, Japan).

Available phosphorus (AP): Measured using the NaHCO₃ extraction-molybdenum antimony anti-colorimetric method (Olsen method). 2.5 g of air-dried soil sample was extracted with 0.5 mol/L NaHCO₃ solution (pH 8.5) at a shaking speed of 180 r/min for 30 min. The supernatant was filtered, and AP content was determined colorimetrically at 700 nm following the same color development procedure as TP.

Total potassium (TK): Determined via the NaOH fusion-flame photometric method. 0.25 g of air-dried soil sample was fused with NaOH in a nickel crucible at 720 °C for 15 min, and the melt was dissolved, filtered, and fixed to volume. TK content in the filtrate was measured using a flame photometer (FP6410, INESA, China).

Available potassium (AK): Measured using the ammonium acetate extraction-flame photometric method. 5.0 g of air-dried soil sample was extracted with 1.0 mol/L neutral NH₄OAc solution at 180 r/min for 30 min. The supernatant was filtered, and AK content was determined using the same flame photometer as TK.

Total calcium (Ca) and total magnesium (Mg): Quantified by inductively coupled plasma optical emission spectrometry (ICP-OES, iCAP 7,400, Thermo Fisher, United States). 0.2 g of air-dried soil sample was digested with a mixed acid system (HNO₃-HClO₄-HF, volume ratio 5:1:1) on a temperature-controlled hot plate. The detailed digestion procedure was as follows: the sample was pre-digested at 120 °C for 2 h, then heated to 180 °C until the digestion solution was clear, and the residual acid was evaporated to near dryness. The digest was diluted to a constant volume of 25 mL with 2% HNO₃ solution and filtered through a 0.45 μm filter membrane before instrumental determination.

### Soil enzyme activities analysis

All enzyme activity assays were performed using kits provided by Solarbio Science & Technology Co., Ltd. (Beijing, China). The brief procedure was as follows: 0.1 g of sample was weighed and homogenized with 1 mL of provided buffer. The mixture was incubated in a water bath at 37 °C for 15 min according to the kit’s instructions. The reaction products were measured using a microplate reader (Epoch2, BioTek, Winooski, United States). The specific kits used were: urease (S-UE, BC0120), catalase (S-CAT, BC0100), alkaline phosphatase (S-ALP/AKP, BC0285), and sucrase (S-SC, BC0245). The specific kits used were: urease (S-UE, BC0120), catalase (S-CAT, BC0100), alkaline phosphatase (S-ALP/AKP, BC0285), and sucrase (S-SC, BC0245). The corresponding units were μg/d/g soil for urease, mM/d/g soil for catalase, μM/d/g soil for alkaline phosphatase, and mg/d/g soil for sucrase.

### Soil high-throughput sequencing

Rhizosphere soil samples were collected at 90 days after planting in each crop cycle. Genomic DNA was extracted from the samples using the MagBeads FastDNA Kit for Soil (MP Biomedicals, USA) and quantified spectrophotometrically. The bacterial 16S rRNA gene and the fungal ITS1 region were amplified with specific primers. The PCR products were quantified using the Quant-iT PicoGreen dsDNA Assay Kit on a Microplate Reader (BioTek, FLx800) and then pooled according to the required data volume for each sample. Libraries were constructed using the TruSeq Nano DNA LT Library Prep Kit (Illumina), and after library quality inspection, paired-end sequencing (2 × 250 bp) was performed on an Illumina NovaSeq platform using the NovaSeq 6000 SP Reagent Kit (500 cycles).

### Data analysis

Statistical analyses of plant phenotypic traits and soil physicochemical properties were performed using SPSS 26.0 (IBM, Chicago, IL, USA). One-way analysis of variance (ANOVA) followed by Duncan’s multiple range test was used to compare differences in plant phenotypic traits, soil physicochemical properties, and enzyme activities among treatments, with a significance threshold of *p* < 0.05. Graphs were generated using GraphPad Prism 9.0.

For microbial data, alpha diversity indices (Chao1, Shannon, Simpson, and Pielous_e) were compared using the Kruskal–Wallis test. Beta diversity was assessed via principal coordinate analysis (PCoA) based on Bray–Curtis distances. The relative abundances of dominant microbial genera were compared using the Kruskal–Wallis test, and linear discriminant analysis effect size (LEfSe) analysis (LDA score ≥ 3.0) was performed to identify differential microbial biomarkers.

To evaluate relationships between microbial communities and environmental factors, Spearman correlation analysis was conducted to assess associations between dominant microbial genera and soil properties/plant phenotypes. Redundancy analysis (RDA), variance partitioning analysis (VPA), and Mantel tests were applied to quantify the driving effects of environmental factors on microbial community structure. A petal plot (flower plot) was used to visualize core and unique operational taxonomic units (OTUs) across treatments. All bioinformatics analyses were completed on the Genescloud platform.

## Results

### Effects of crop rotation systems on agronomic traits of cut Chrysanthemums

**Figure 1:**
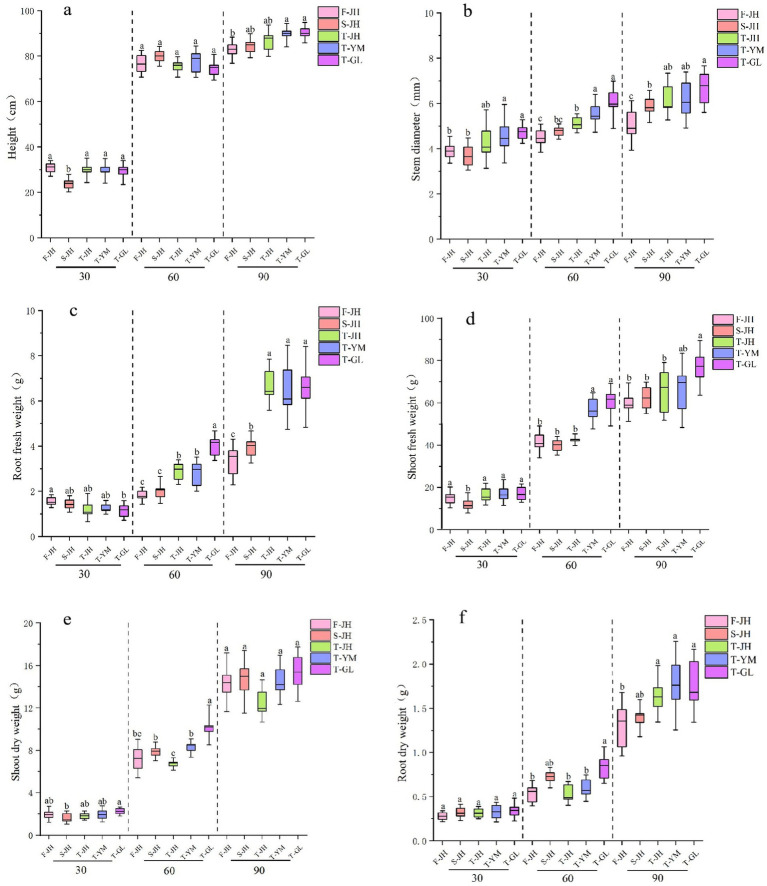
Phenotypic traits of cut chrysanthemum under different treatments: **(a)** Plant height; **(b)** stem diameter; **(c)** fresh root weight; **(d)** fresh stem weight; **(e)** dry stem weight; **(f)** dry root weight. F-JH, First crop of chrysanthemum group; S-JH, Second crop of chrysanthemum group; T-JH, Third crop of chrysanthemum group; T-YM, Third crop following maize rotation; T-GL, Third crop following cabbage rotation. Numbers 30, 60, and 90 represent the number of planting days. Different lowercase letters indicate significant differences (*p* < 0.05), and error bars represent standard deviations.

At the early vegetative growth stage (30 days after planting), no significant differences in any of the above phenotypic indices were observed among the three treatments (Figures 1a–f). The growth disparities between treatments became pronounced at the mid-growth stage (60 days, early reproductive growth stage of the third crop), with the cabbage rotation group exhibiting the optimal overall growth performance. At this stage, both the cabbage and maize rotation systems resulted in significantly greater stem diameter, fresh stem weight, and dry stem weight compared to the continuous cropping control, with increases ranging from 10.3% to 51% (Figures 1b,d,e). Further inter-group comparison showed that the fresh root weight and dry root weight in the cabbage rotation group were 45.6 and 41% higher than those in the continuous cropping group at 60 days of the third crop, respectively, reaching a statistically significant level (Figures 1c,f). By the later growth stage (after 90 days, the commercial harvesting phase), the differences in most phenotypic indicators among the three treatment groups had narrowed. Although the rotation groups still exhibited higher values across all growth indicators, most of the differences were no longer statistically significant. However, the cabbage rotation group retained significantly greater shoot fresh weight compared with the continuous cropping group (Figure 1d).

### Effects of different treatments on soil physicochemical properties

**Figure 2:**
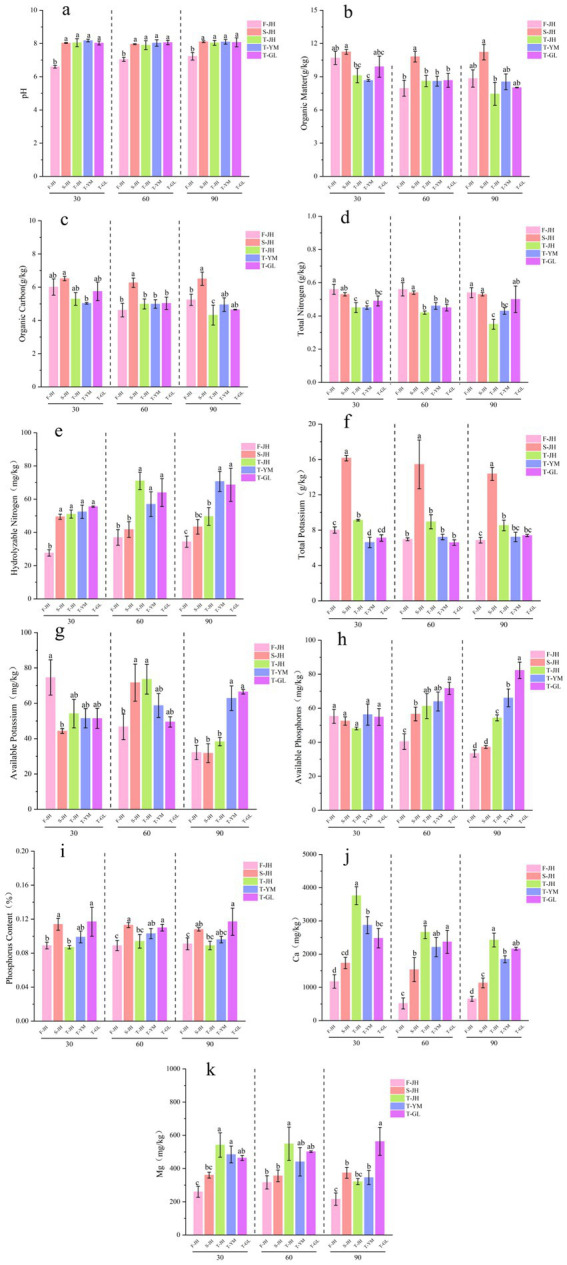
Soil physicochemical properties under different treatments: **(a)** pH; **(b)** Organic matter; **(c)** Organic carbon; **(d)** Total nitrogen; **(e)** Hydrolyzable nitrogen; **(f)** Total potassium; **(g)** Available potassium; **(h)** Available phosphorus; **(i)** Phosphorus content; **(j)** Calcium (Ca); **(k)** Magnesium (Mg). F-JH, First crop of chrysanthemum group; S-JH, Second crop of chrysanthemum group; T-JH, Third crop of chrysanthemum group; T-YM, Third crop following maize rotation; T-GL, Third crop following cabbage rotation. Here, 30, 60, and 90 represent the number of planting days. Different lowercase letters indicate significant differences (*p* < 0.05), and error bars represent standard deviations.

The dynamic changes in key soil properties, including pH, organic matter, nitrogen (total and hydrolyzable), phosphorus (total and available), potassium (total and available), calcium (Ca), and magnesium (Mg), under different treatments are shown in Figure 2. Soil pH increased significantly at the beginning of the second crop and remained relatively stable thereafter (Figure 2a), indicating that the second crop represents a critical transition period for soil pH.

Regarding temporal dynamics, soil organic matter content gradually decreased across all treatments with successive cropping, while the contents of hydrolyzable nitrogen and available potassium exhibited a general increasing trend. Crucially, the crop rotation systems led to significant improvements in several key nutrients. Specifically, the contents of soil total nitrogen, hydrolyzable nitrogen, available phosphorus, and phosphorus in both rotation treatments were consistently higher than those in the continuous cut chrysanthemum cropping group.

The temporal analysis of soil nutrients revealed distinct treatment effects. At 30 days after planting, no significant differences in soil nutrient contents were detected among the three treatments. However, the advantages of rotation became pronounced by the late stage (90 days) of the third crop. Specifically, the maize and cabbage rotation groups exhibited significantly higher levels of available potassium and organic carbon than the continuous cropping group. Notably, the contents of available potassium and organic carbon in the maize and cabbage rotation groups were significantly higher than those in the continuous cropping group (Figures 2c,g). Furthermore, at 90 days, the contents of total nitrogen, hydrolyzable nitrogen, and available phosphorus in the cabbage rotation group were all significantly higher than those in the continuous cropping group (Figures 2d,e,h). The maize rotation group also registered significant increases in hydrolyzable nitrogen and available phosphorus (Figures 2e,h). Notably, the cabbage rotation group maintained the highest levels of both total and available phosphorus throughout the entire growth period of the third crop (Figures 2h,i), underscoring its pronounced effect on enhancing soil phosphorus accumulation and availability.

**Figure 3:**
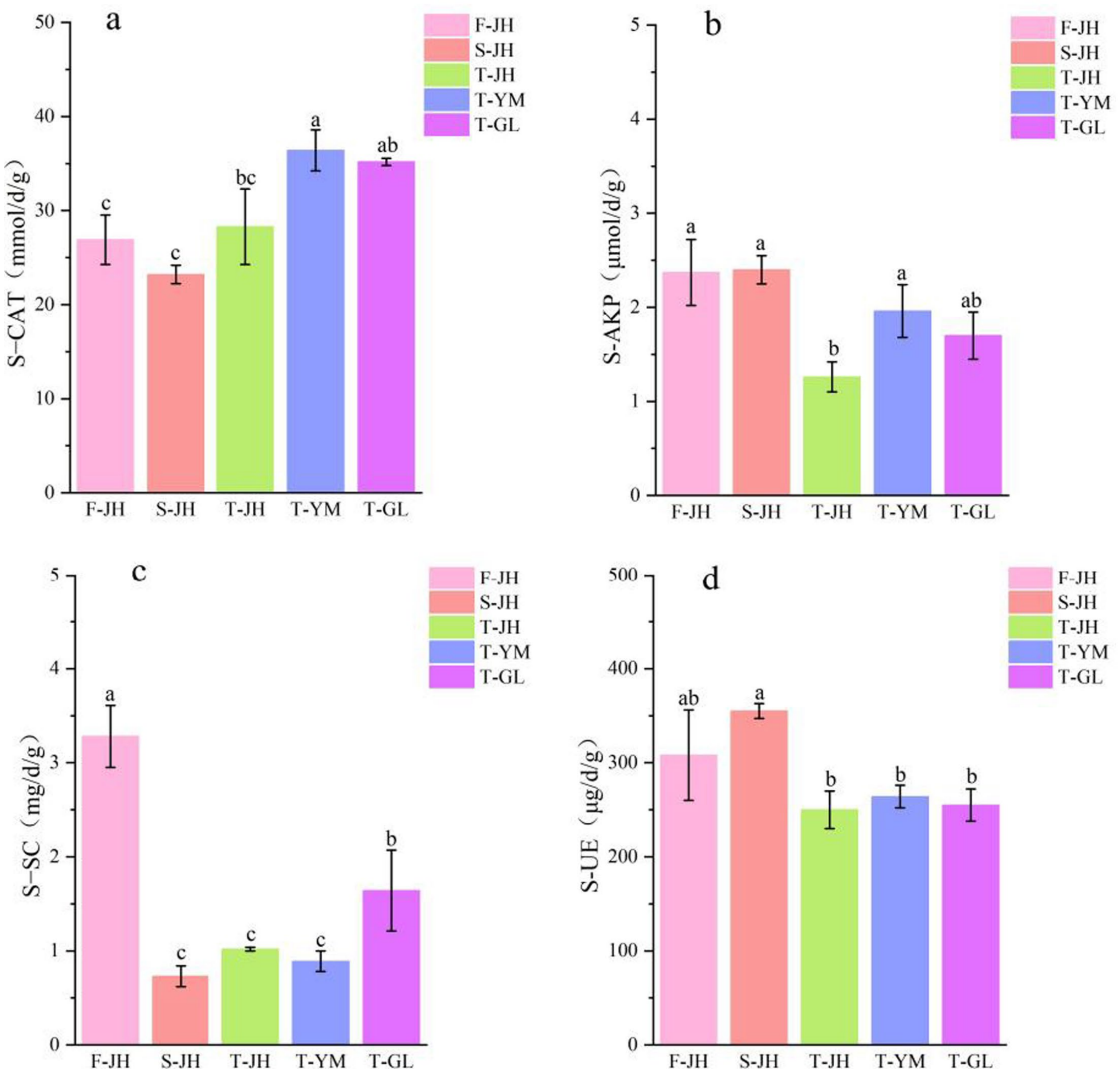
Soil enzyme activities under three treatments. **(a)** Catalase; **(b)** alkaline phosphatase; **(c)** sucrase; **(d)** urease. F-JH, First crop of chrysanthemum group; S-JH, Second crop of chrysanthemum group; T-JH, Third crop of chrysanthemum group; T-YM, Third crop following maize rotation; T-GL, Third crop following cabbage rotation. Different lowercase letters indicate significant differences (*p* < 0.05), and error bars represent standard deviations.

The treatments also significantly influenced soil enzyme activities. Overall, the activities of most soil enzymes, except for catalase, decreased with successive cropping cycles. Despite this general trend, the rotation treatments effectively enhanced specific soil enzyme activities. Specifically, the activities of catalase and alkaline phosphatase in the maize rotation group were significantly higher than those in the continuous cropping group. Meanwhile, the cabbage rotation group exhibited the highest sucrase activity, which was significantly greater than that in both the maize rotation group and the continuous cropping group (Figures 3a–d).

### Crop rotation systems alter the diversity of bacterial and fungal communities

**Figure 4:**
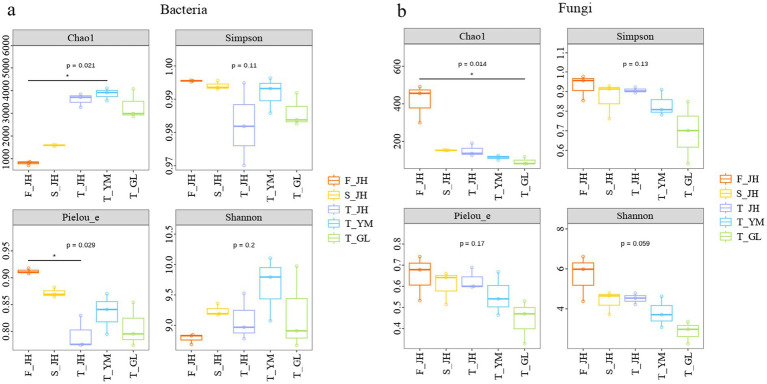
Bacterial **(a)** and fungal **(b)** *α*-diversity under three treatments. F-JH, First crop of chrysanthemum group; S-JH, Second crop of chrysanthemum group; T-JH, Third crop of chrysanthemum group; T-YM, Third crop following maize rotation; T-GL, Third crop following cabbage rotation. * indicates *p* < 0.05.

We systematically analyzed the *α*-diversity characteristics of rhizosphere bacterial and fungal communities of cut chrysanthemums under three treatments to reveal how treatments and planting duration shape their structure (Figure 4). A key temporal trend was observed: bacterial community α-diversity increased with successive planting cycles, whereas fungal diversity exhibited a continuous decline.

Regarding bacterial α-diversity (Figure 4a), within the same crop (the third crop), the maize rotation group exhibited the highest values for all indices. Its Chao1 index was significantly greater than that of the first crop of continuous chrysanthemum cropping group. In addition, the Pielous_e index of the third continuous chrysanthemum cropping group was significantly lower than that of the first, indicating that long-term continuous cropping would reduce the evenness of bacterial communities. In contrast, crop rotation could effectively improve the richness and overall diversity level of bacterial communities.

For fungal community α-diversity (Figure 4b), the Chao1 index of the first continuous chrysanthemum cropping group was significantly higher than that of the cabbage rotation group, while no statistically significant differences were observed among the treatment groups for other α-diversity indices such as Shannon, Simpson and Pielous_e.

A direct comparison between rotation and continuous treatments within the same crop cycle revealed distinct microbial patterns. The rhizosphere bacterial richness of rotation treatments (maize rotation and cabbage rotation) was significantly higher than that of the continuous cropping group, but lower fungal richness compared to continuous cropping group. Moreover, the rotation groups exhibited higher bacterial community evenness. Collectively, these results demonstrate that crop rotation optimizes the structure of the rhizosphere microbial community by simultaneously enhancing bacterial diversity and suppressing fungal proliferation.

**Figure 5:**
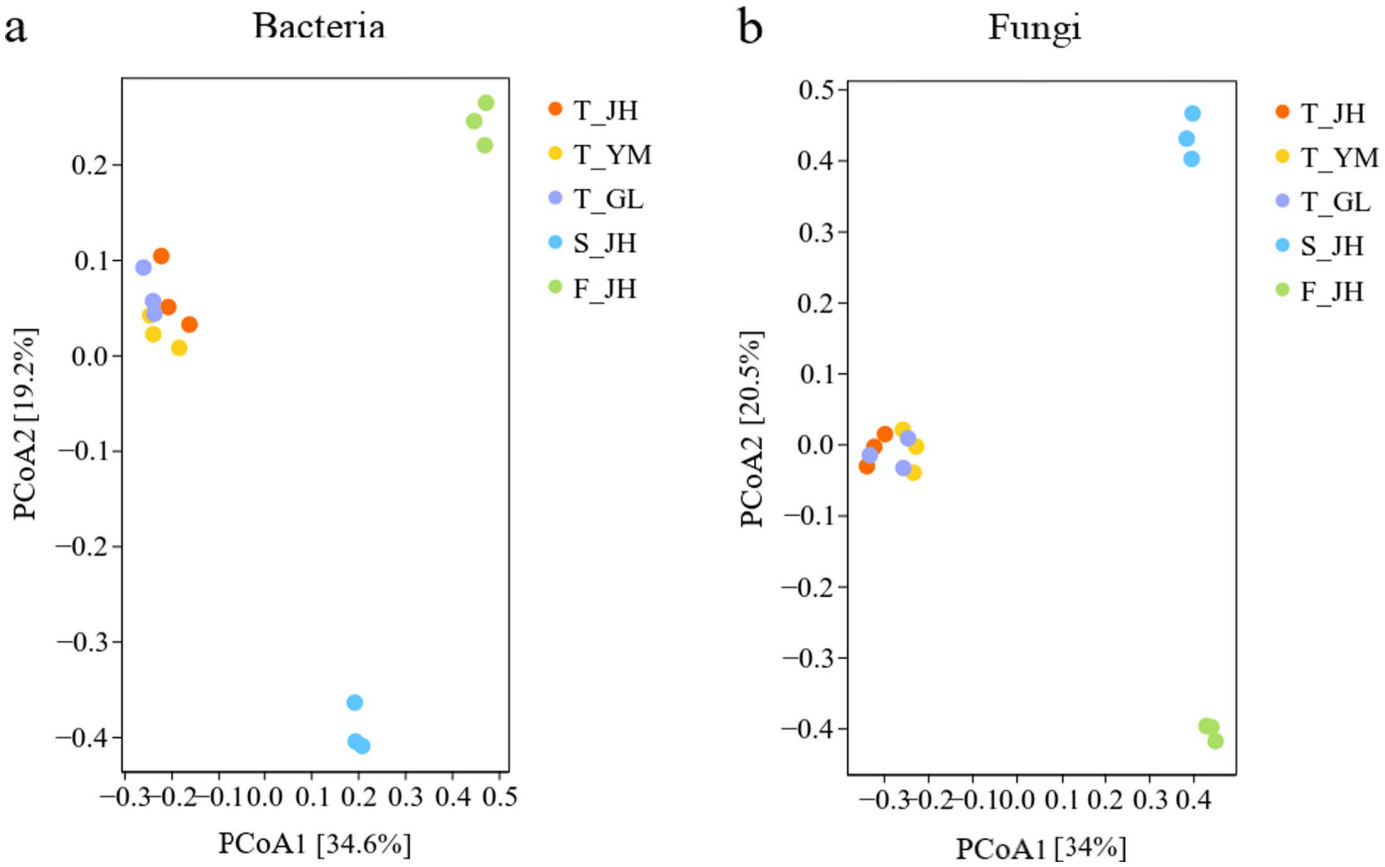
Principal coordinate analysis (PCoA) of bacteria **(a)** and fungi **(b)** under three treatments. F-JH, First crop of chrysanthemum group; S-JH, Second crop of chrysanthemum group; T-JH, Third crop of chrysanthemum group; T-YM, Third crop following maize rotation; T-GL, Third crop following cabbage rotation.

To assess the divergence in rhizosphere microbial community structures among the treatments, we conducted a principal component analysis (PCA) based on Bray-Curtis distances (Figure 5). In the ordination plot for bacterial communities (Figure 5a), distinct clustering was observed according to the three planting patterns. The variance explanation rates of the first principal component (PC1) and the second principal component (PC2) were 34.6 and 19.2% respectively, with a cumulative variance explanation rate of 53.8%, which could well reflect the main differentiation information of bacterial community structures. Similarly, fungal communities (Figure 5b) also exhibited clear separation by treatment. The variance explanation rate of the first principal component (PC1) was 34%, and that of the second principal component (PC2) was 20.5%, with a cumulative variance explanation rate of 54.5%, which could effectively reveal the main sources of variation in fungal community structures.

**Figure 6:**
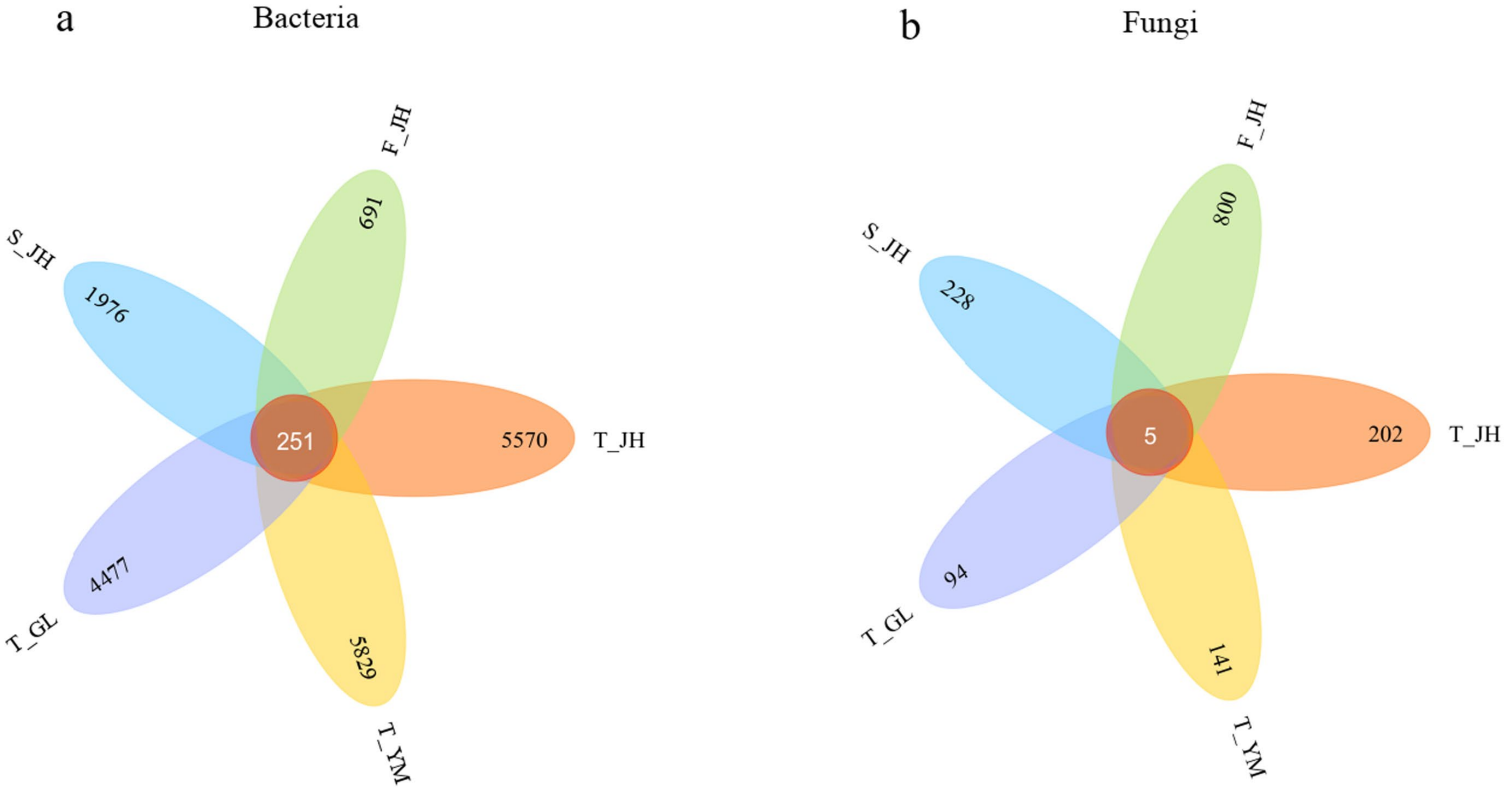
Petal plots of bacteria **(a)** and fungi **(b)** under three treatments. F-JH, First crop of chrysanthemum group; S-JH, Second crop of chrysanthemum group; T-JH, Third crop of chrysanthemum group; T-YM, Third crop following maize rotation; T-GL, Third crop following cabbage rotation.

The petal plot analysis (Figure 6) clarified the number of operational taxonomic units (OTUs) and the distribution of shared and unique OTUs in the rhizosphere bacterial and fungal across different crop cycles and planting patterns. This analysis reveals the core and habitat-specific microbiota, thereby informing the differentiation patterns of microbial community composition.

Analysis of rhizosphere bacterial communities revealed distinct patterns in OTU distribution (Figure 6a). The number of unique OTUs was 691 in the first continuous crop, 1,976 in the second, and 5,570 in the third. Notably, the third crop under maize and cabbage rotations harbored 5,829 and 4,477 unique OTUs, respectively. A core microbiome of 251 bacterial OTUs was shared across all treatments. From the perspective of the overall variation trend, the total number of bacterial OTUs increased significantly with successive cropping cycles, reflecting a gradual accumulation of bacterial richness over time. Furthermore, within the same crop cycle (third crop), the maize rotation group had the highest number of bacterial OTUs, significantly higher than both continuous cropping group and cabbage rotation group. This further confirms that the maize rotation pattern has a better effect on improving the species diversity of the rhizosphere bacterial community.

In contrast to the bacterial community, the rhizosphere fungal OTUs exhibited a divergent pattern (Figure 6b). The number of unique fungal OTUs declined substantially across treatments: 800 in the first continuous crop, 228 in the second, and 202 in the third. This decreasing trend was further amplified under rotation, with the maize and cabbage rotations in the third crop showing only 141 and 94 unique OTUs, respectively. The shared core of fungal OTUs across all groups was remarkably small, which was significantly lower than the number of shared bacterial OTUs. This indicates that the rhizosphere fungal community has stronger specificity in species composition and fewer shared core species. Temporally, the total number of fungal OTUs showed a significant downward trend with successive cropping, reflecting the inhibitory effect of long-term cultivation on fungal richness. Among the third-crop treatments, the cabbage rotation resulted in the lowest fungal OTUs count, suggesting that the cabbage rotation pattern has the most significant effect on reducing the species diversity of the rhizosphere fungal community.

### Rhizosphere microbial composition shifts under different treatments

To elucidate the impact of continuous cropping and rotation on rhizosphere microbial communities, we analyzed the relative abundance of dominant microbial taxa across the three treatments.

**Figure 7:**
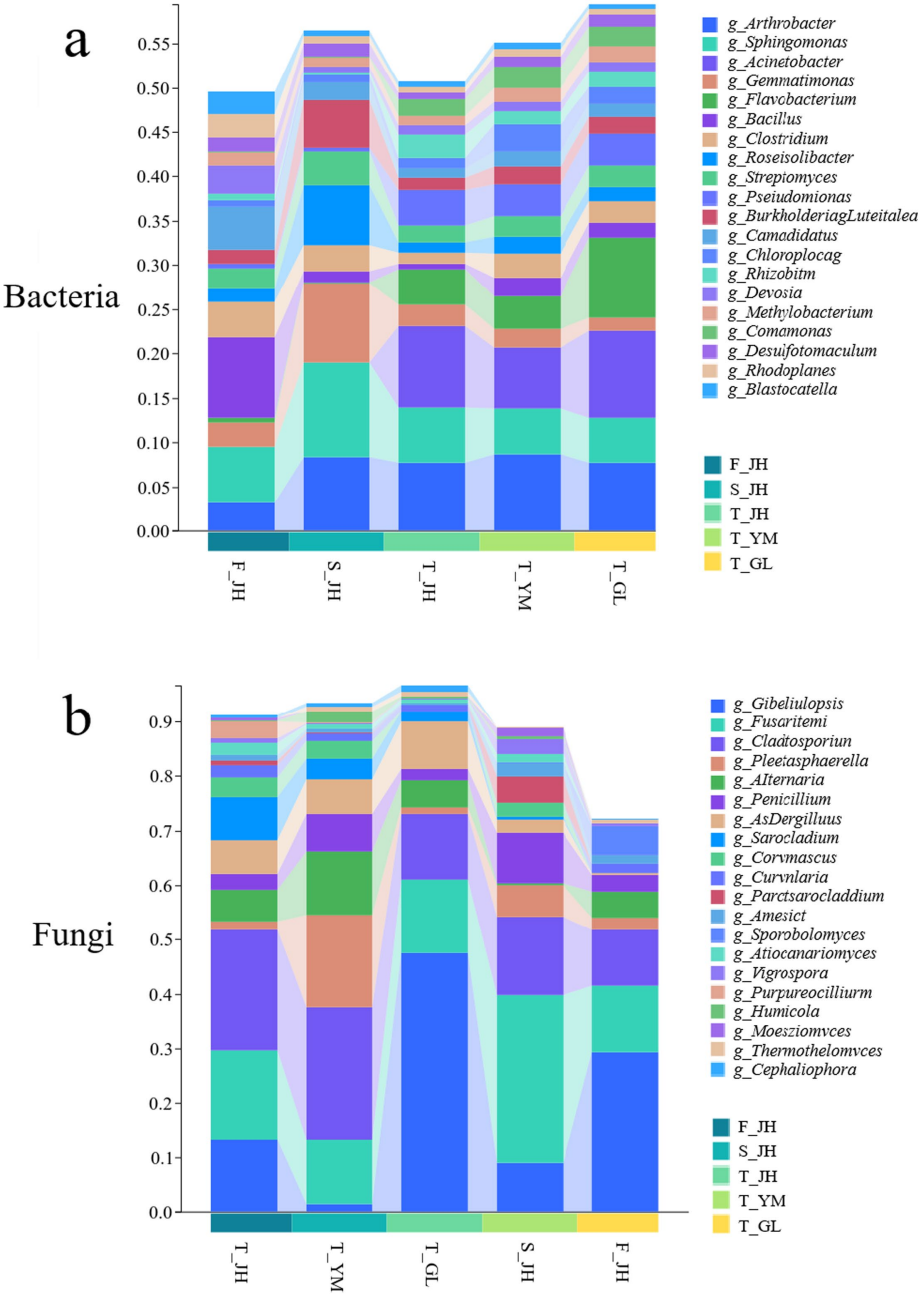
Relative abundance of the top 20 genera of bacteria **(a)** and fungi **(b)** under three treatments. F-JH, First crop of chrysanthemum group; S-JH, Second crop of chrysanthemum group; T-JH, Third crop of chrysanthemum group; T-YM, Third crop following maize rotation; T-GL, Third crop following cabbage rotation.

The composition of dominant bacterial genera varied considerably across treatments (Figure 7a). Genera such as *Arthrobacter*, *Sphingomonas*, and *Acinetobacter* were consistently abundant in all treatments, serving as the core shared dominant taxa in the rhizosphere bacterial community. Their relative abundances, however, fluctuated with successive cropping cycles. Notably, *Flavobacterium* (TOP5) was significantly enriched in the third crop cabbage group, *Candidatus chloroploca* (TOP13) in the third crop maize group, and *Rhizobium* (TOP14) in the third crop chrysanthemum group. Temporal trends revealed that the abundances of *Acinetobacter* and *Flavobacterium* increased significantly over time, while the abundance of *Gemmatimonas* decreased. Compared with continuous cropping in the same crop cycle, the relative abundance of *Bacillus* was lower under rotation, whereas that of *Rhizobium* was higher.

Several fungal genera such as *Gibellulopsis*, *Fusarium*, and *Cladosporium* exhibited high relative abundances across different treatments (Figure 7b), forming the core shared dominant taxa in the rhizosphere fungal community. Their relative abundances shifted markedly across crop cycles: *Cephalotheca* was significantly enriched in the third crop cabbage group, and *Humicola* in the third crop maize group. Among these, the abundances of *Fusarium* and *Cladosporium* increased significantly with successive cropping, whereas the abundance of *Alternaria* decreased. Compared to continuous cropping in the same crop cycle, crop rotation systems supported lower relative abundances of genera such as *Gibellulopsi*s. Notably, the abundance of pathogenic fungus *Fusarium* was the highest in the third crop continuous cropping group, and the *Fusarium* contents in the two rotation groups were 28.1 and 17.1% lower than that, respectively.

**Figure 8:**
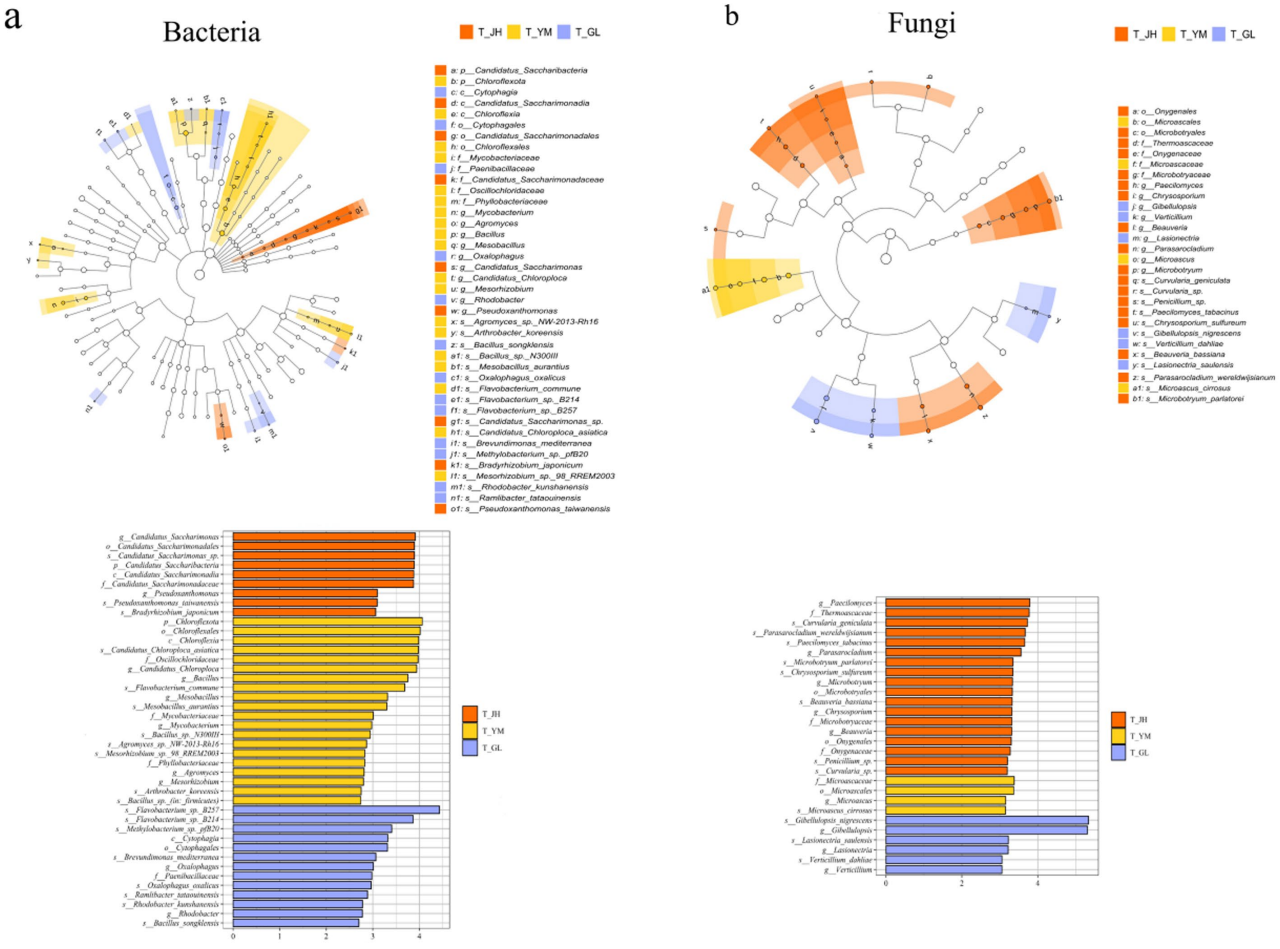
LEfSe analysis and LDA bar charts of bacteria **(a)** and fungi **(b)** under three treatments. T-JH, Third crop of chrysanthemum group; T-YM, Third crop following maize rotation; T-GL, Third crop following cabbage rotation.

**Figure 9:**
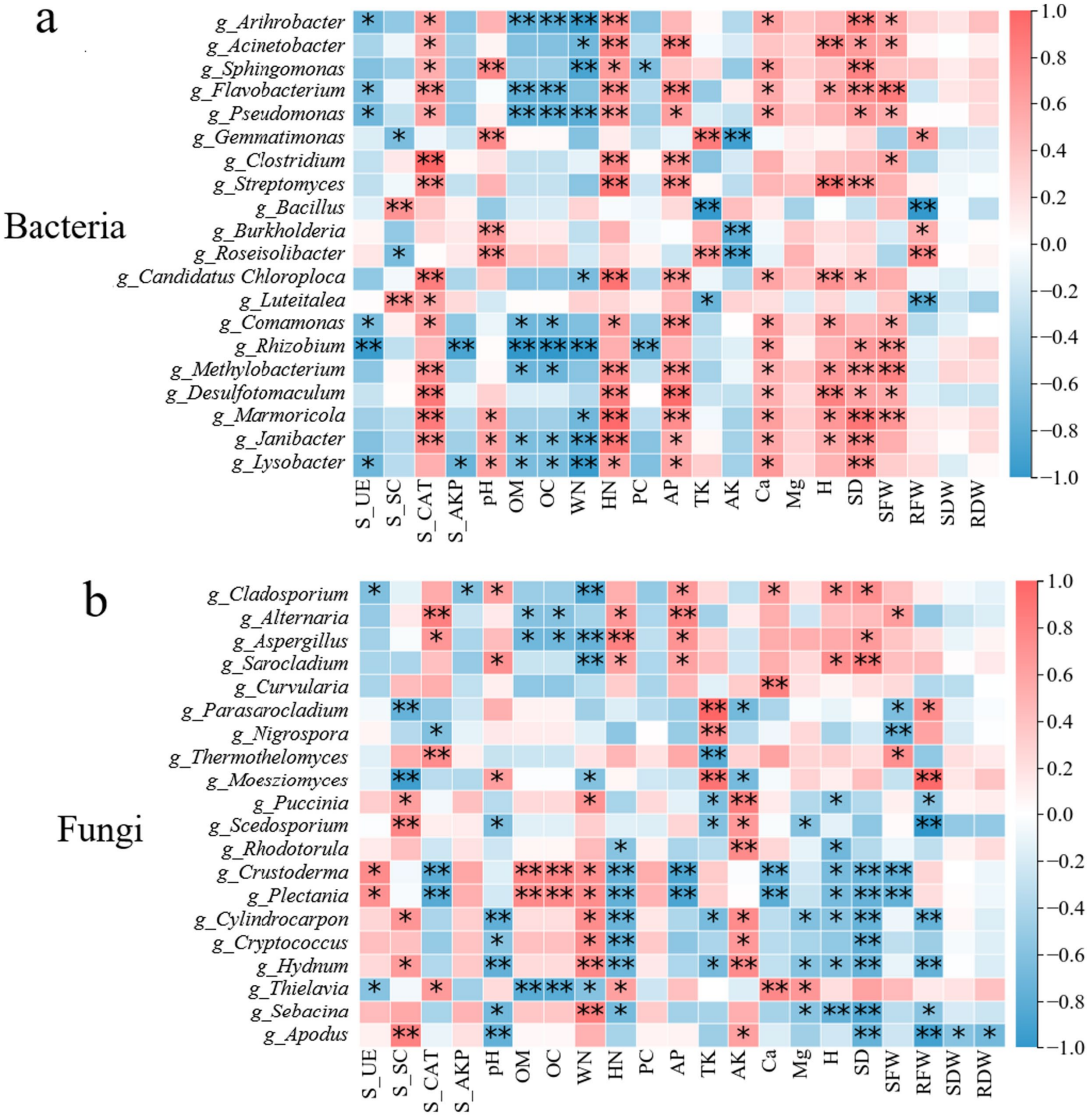
Heatmaps of correlations between bacteria **(a)** / fungi **(b)** and soil physicochemical properties, plant phenotypes under three treatments. S_UE, Urease activity; S_SC, Sucrase activity; S_CAT, Catalase activity; S_AKP, Alkaline phosphatase activity; OM, Organic matter; OC, Organic carbon; WN, Total nitrogen; HN, Hydrolyzable nitrogen; PC, Phosphorus content; AP, Available phosphorus; TK, Total potassium; AK, Available potassium; Ca, Calcium; Mg, Magnesium; H, Plant height; SD, Stem diameter; SFW, Fresh stem weight; SRW, Fresh root weight; SDW, Dry stem weight; RDW, Dry root weight. * indicates *p* < 0.05, ** indicates *p* < 0.01.

To identify microbial taxa significantly enriched under different cropping systems, we performed LEfSe analysis on the third crop of continuous chrysanthemum, maize rotation, and cabbage rotation groups. For bacterial community (Figure 8a), 9, 20, and 13 biomarkers were identified for the continuous chrysanthemum, maize rotation, and cabbage rotation, respectively. Among them, the biomarkers of the third crop chrysanthemum group included *Candidatus Saccharibacteria*, the genus *Pseudoxanthomonas* (and its species *P. taiwanensis*), and *Bradyrhizobium japonicum*. In contrast, the biomarkers of maize rotation group included *Bacillus* and its subordinate species, the *Mesorhizobium* and *Chloroflexota*, etc. The biomarkers cabbage rotation group featured distinct biomarkers like the family *Paenibacillaceae* and the genus *Rhodobacter*.

In the fungal community (Figure 8b), LEfSe analysis identified 16, 4, and 6 significant biomarkers for the third crop continuous chrysanthemum, maize rotation, and cabbage rotation groups, respectively. The continuous chrysanthemum group, which contained the highest number of biomarkers, included known taxa such as *Curvularia geniculat*a and *Paecilomyces*. The maize rotation group was characterized by biomarkers including *Microascus*, while the cabbage rotation group featured taxa such as *Lasionectria*.

### Relationship between microbial composition and environmental factors

To elucidate the linkages between soil physical and chemical properties, plant phenotypes, and microorganisms, we performed correlation analysis between microbial relative abundance and environmental factors. The results showed that catalase activity, hydrolyzable nitrogen, phosphorus content, calcium (Ca), plant height, stem diameter, and dry stem weight were significantly positively correlated with abundance of numerous bacterial taxa, including *Arthrobacter*, *Streptomyces* and *Methylobacterium*. In contrast, urease activity, organic matter, organic carbon, and total nitrogen were significantly negatively correlated with bacteria such as *Arthrobacter*, *Pseudomonas*, and *Rhizobium* (Figure 9a).

Fungal taxa exhibited distinct correlation patterns with environmental factors. Organic matter and organic carbon were significantly negatively correlated with *Alternaria* and *Aspergillus*, but significantly positively correlated with *Crustoderma* and *Plectania*. Notably, total nitrogen and hydrolyzable nitrogen showed opposite correlation trends with fungi: fungi that were positively correlated with total nitrogen (e.g., *Aspergillus*, *Sarocladium*) were predominantly negatively correlated with hydrolyzable nitrogen (Figure 9b).

**Figure 10:**
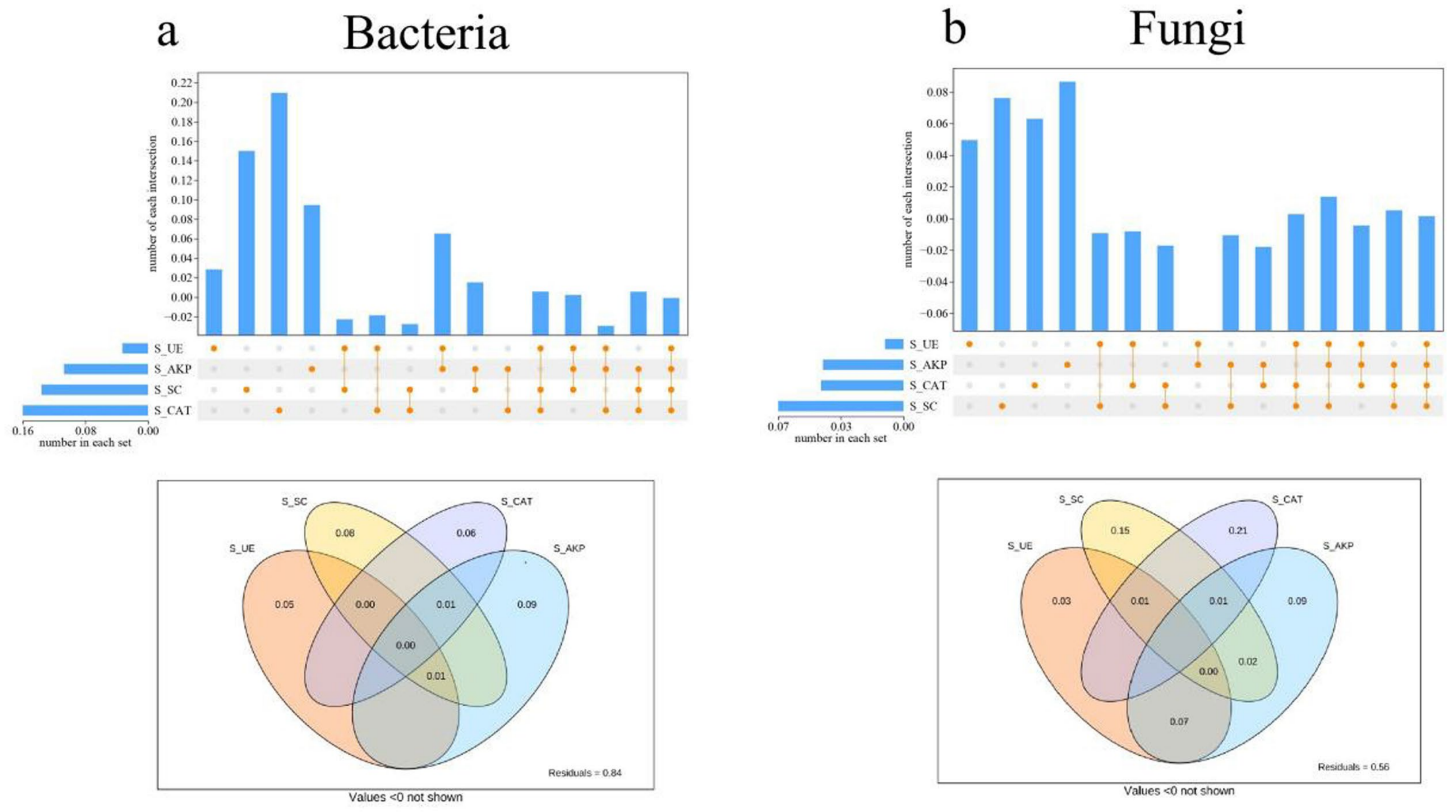
Variance partitioning analysis (VPA) and hierarchical analysis of bacteria **(a)** and fungi **(b)** with soil enzyme activities and microbial communities under three treatments. S_UE, Urease activity; S_SC, Sucrase activity; S_CAT, Catalase activity; S_AKP, Alkaline phosphatase activity.

The relative influence of soil enzyme activities on microbial community structure was assessed using variance partitioning analysis (VPA). The results indicated that catalase activity was the predominant factor shaping the bacterial community, followed by sucrase activity, while the individual and combined effects of other enzymes were minimal (Figure 10a). Regarding the fungal community, alkaline phosphatase activity is the most influential factor, with sucrase, catalase, and urease activities exhibiting comparable but lesser effects. The combined explanatory power of multiple enzymes was also limited for fungi (Figure 10b).

**Figure 11:**
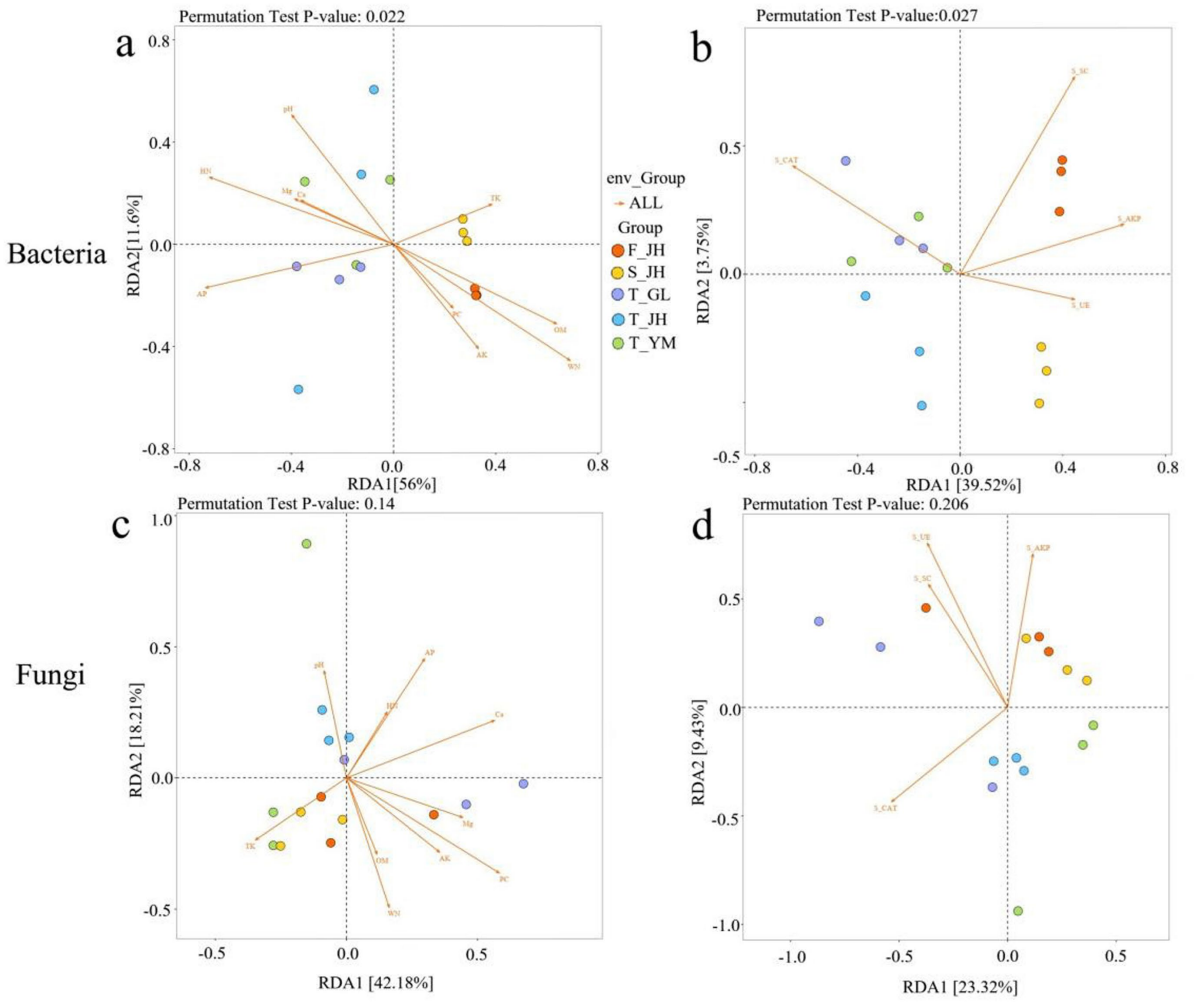
Redundancy analysis (RDA) analysis of bacteria **(a,b)** and fungi **(c,d)** with soil enzyme activities and microbial communities under three treatments. S_UE, Urease activity; S_SC, Sucrase activity; S_CAT, Catalase activity; S_AKP, Alkaline phosphatase activity; OM, Organic matter; OC, Organic carbon; WN, Total nitrogen; HN, Hydrolyzable nitrogen; PC, Phosphorus content; AP, Available phosphorus; TK, Total potassium; AK, Available potassium; Ca, Calcium; Mg, Magnesium; H, Plant height; SD, Stem diameter; SFW, Fresh stem weight; SRW, Fresh root weight; SDW, Dry stem weight; RDW, Dry root weight. * indicates *p* < 0.05, ** indicates *p* < 0.01.

Redundancy analysis (RDA) was employed to quantify the influence of soil properties on bacterial communities. Among soil elements, total nitrogen was the most influential factor (r^2^ = 0.646), followed by hydrolyzable nitrogen, available phosphorus, organic matter, and organic carbon, indicating that nitrogen, phosphorus, and carbon elements are key determinants of bacterial communities (Figure 11a). In a separate RDA focusing on enzyme activities (Figure 11b), sucrase activity was the most influential factor (r^2^ = 0.555), followed by catalase, alkaline phosphatase, and urease activities (Figure 11b).

Redundancy analysis (RDA) was also applied to fungal communities. Among soil elements, phosphorus content exerted the strongest influence (r^2^ = 0.409), followed by calcium, available phosphorus, total nitrogen, and magnesium. It is evident that phosphorus, calcium, magnesium, and nitrogen elements are important factors affecting fungal communities (Figure 11c). In the RDA analysis of enzyme activities, catalase activity was the most influential factor (r^2^ = 0.363), ahead of alkaline phosphatase, urease, and sucrase (Figure 11d).

**Figure 12:**
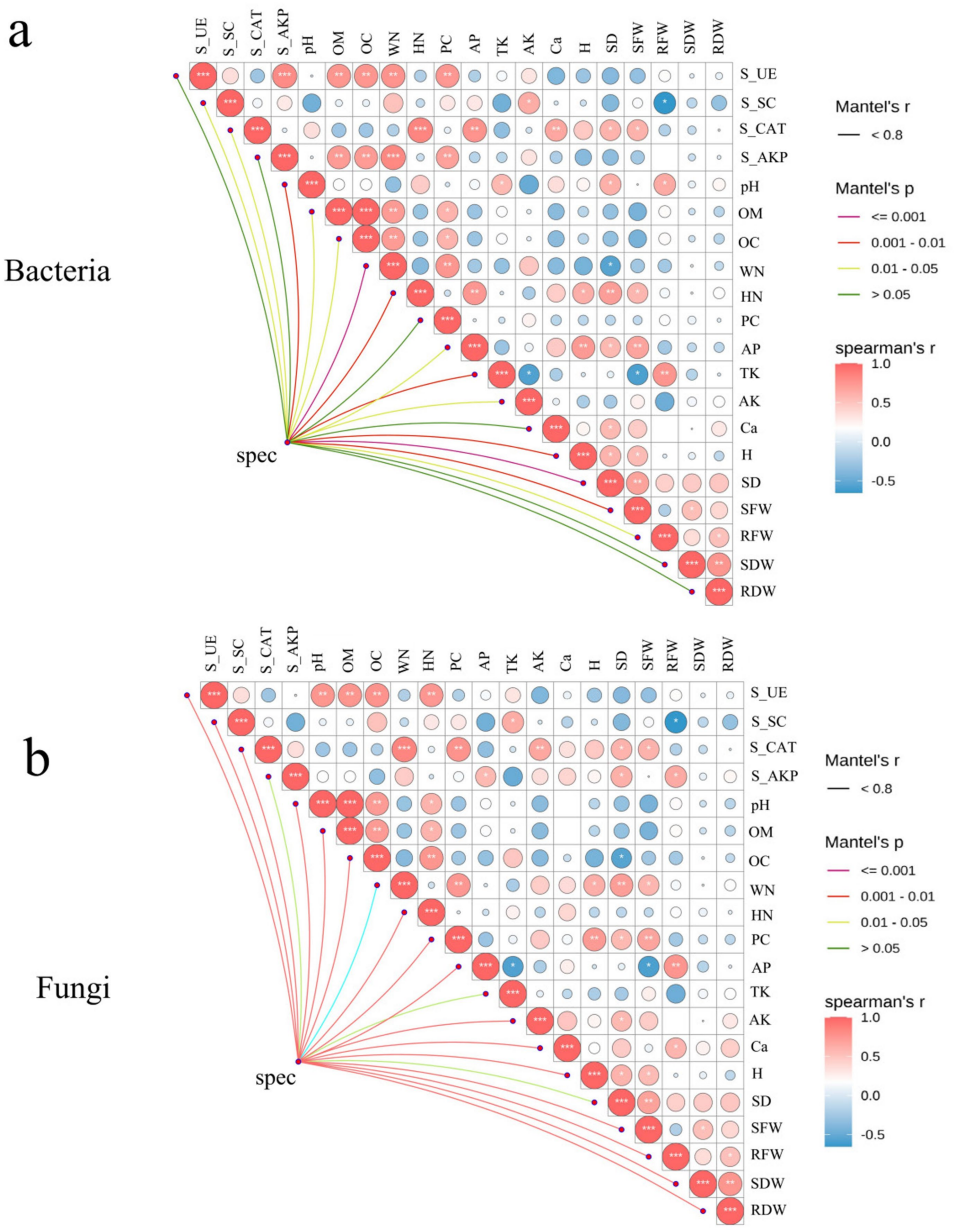
Mantel test analysis of bacteria **(a)** and fungi **(b)** with soil enzyme activities and microbial quantities under three treatments. S_UE, Urease activity; S_SC, Sucrase activity; S_CAT, Catalase activity; S_AKP, Alkaline phosphatase activity; OM, Organic matter; OC, Organic carbon; WN, Total nitrogen; HN, Hydrolyzable nitrogen; PC, Phosphorus content; AP, Available phosphorus; TK, Total potassium; AK, Available potassium; Ca, Calcium; Mg, Magnesium; H, Plant height; SD, Stem diameter; SFW, Fresh stem weight; SRW, Fresh root weight; SDW, Dry stem weight; RDW, Dry root weight. * indicates *p* < 0.05, ** indicates *p* < 0.01.

To gain a deeper understanding of the relationships between microorganisms and various factors, we performed a Mantel test analysis. The results demonstrated that the bacterial community had significant correlations with all factors except urease, alkaline phosphatase, phosphorus content, calcium, dry stem weight, and dry root weight (Figure 12a), indicating that the external environment has a significant impact on the bacterial community. In contrast, fungi showed significant correlations only with pH, hydrolyzable nitrogen, available potassium, and stem diameter (Figures 12b).

## Discussion

### Effects of different rotation patterns on growth, quality, and soil physicochemical properties of cut chrysanthemums

Continuous monoculture often leads to reduced yield, increased incidence of diseases and pests, and decreased quality. Crop rotation serves as a key agronomic measure to mitigate continuous cropping obstacles by promoting plant growth and suppressing soil-borne diseases (Liu et al., 2025; Wang et al., 2025). In this study, we observed that at 60 days after transplanting (early reproductive growth stage), stem diameter, fresh stem weight, and dry stem weight of cut chrysanthemums under rotation were significantly higher than those under continuous cropping. However, by 90 days, only the cabbage rotation group showed significantly greater shoot fresh weight (Figure 1d) compared with the continuous cropping group. This phenomenon might be explained from a compensatory growth mechanism: in the later reproductive stages, continuous cropping plants may reallocate photosynthetic products, prioritizing supply to reproductive organs such as flowers to maintain reproduction, thereby exhibiting partial recovery in final biomass (Zhou et al., 2022; Li et al., 2018). More importantly, the deterioration of the rhizosphere environment induced by continuous cropping, such as increased pathogen abundance and reduced soil enzyme activities, persists throughout the entire growth cycle. This potential production risk cannot be eliminated by compensatory effects on a single biomass index alone.

Plant growth depends strongly on healthy soil conditions, with soil nutrients and enzyme activities being among the most critical factors influencing plant development. Crop rotation has been recognized as an effective practice for maintaining the balance of various soil nutrients and establishing favorable growing conditions for plants (Yang et al., 2024; Zhao et al., 2024; Zhou et al., 2023). Studies have shown that long-term consecutive planting of chrysanthemums leads to a decrease in nitrogen, phosphorus, and potassium content, resulting in soil nutrient imbalance (Liu et al., 2022). Similarly, there are also reports that after growing cut chrysanthemums continuously for 12 years, the soil fertility significant declines (Li et al., 2023a), which is unfavorable for the subsequent growth and development of chrysanthemums. In the present study, although no significant difference in soil pH was detected between rotation and continuous cropping, the contents of soil total nitrogen, hydrolyzable nitrogen, available phosphorus and other nutrients in the continuous cropping group were significantly lower than those in the rotation group, this indicates that crop rotation effectively maintain soil nitrogen and phosphorus elements, thereby preventing nutrient imbalance, which is consistent with previous reports (Yang et al., 2024; Zhao et al., 2024; Zhou et al., 2023). Moreover, we found that the cabbage rotation group had the highest phosphorus content, implying that planting cabbage may possess specific advantages in sustaining or enhancing soil phosphorus availability.

Soil enzymes play a vital role in converting organic matter into plant-available nutrients and are integral to processes such as soil organic matter restoration and soil structure (Memane et al., 2025). In this study, the activities of most key enzymes, except for catalase activity, decreased over time, which is consistent with the research results of Liao et al. (2025). Moreover, on the whole, the enzyme activities were generally higher under the rotation than that under the continuous cropping. This enhancement may be attributed to root exudates from rotation crops, which can stimulate microbial activity and thereby influence the soil enzyme environment, representing a potential mechanism through which rotation supports the growth of cut chrysanthemums.

### Microbial community restructuring under rotation management

The abundance and diversity of soil microorganisms are key indicators of soil health, with a high and stable microbial diversity index being essential for a robust soil ecosystem. Soil microorganisms play a fundamental role in nutrient transformation (Bahram et al., 2018). Our *α*-diversity analysis revealed that bacterial diversity increased with successive crop cycles, whereas fungal diversity declined. This indicates that the soil is changing toward a healthy direction, reflecting the differential evolution law of “enrichment of bacterial diversity - attenuation of fungal diversity” in the rhizosphere microbial community during long-term planting. This phenomenon has also been observed in the long-term cultivation of the cut chrysanthemum ‘Guangyu’ (Liu et al., 2023). This pattern may be driven by the selective pressure exerted on bacterial and fungal communities by long-term changes in soil nutrients and enzyme activities.

Moreover, within the same crop cycle, rotation group yielded the highest values of various bacterial diversity indices in the same crop cycle, while the fungal richness was lower than that in the continuous cropping group, indicating that the soil in the rotation group was healthier, which has been confirmed by multiple reports (Yu et al., 2025; Ma et al., 2024). The observed trends in OTUs for both bacteria and fungi were fully consistent with the α-diversity patterns, providing robust evidence for the synchronous and correlated shifts in these communities. This further confirms that crop rotation exerts a selective and regulatory influence on the rhizosphere microbiota. Analysis of *β* diversity showed that, both fungi and bacteria formed distinct clusters according to the cropping system within the same crop cycle, indicating that the selective regulatory effect of planting patterns on the fungal community structure did not lead to widespread community dispersal but instead resulted in relatively stable, treatment-specific core microbial structures.

Analysis of microbial composition further demonstrated that continuous cropping exerts a significant impact on the relative abundance of microorganisms. Notably, continuous cropping led to the enrichment of bacterial *Pseudoxanthomonas* and the *Bradyrhizobium japonicum* in rhizosphere of cut chrysanthemums. Literature reports indicate that the *Pseudoxanthomonas* plays an important role in suppressing *Fusarium oxysporum* and exhibits activity against root-knot nematodes (Pheirim and Sobita, 2024; Shakhnazarova et al., 2023; Hu et al., 2019). Meanwhile, *Bradyrhizobium japonicum* is one of the effective biocontrol agents against soybean charcoal rot and tomato bacterial wilt, and it can also increase soybean biomass and nodule formation (Umar et al., 2024; Dabré et al., 2022; Chattopadhyay et al., 2022).

In the maize rotation group, the rhizosphere was significantly enriched with the genus *Bacillus*, which is widely recognized for its broad-spectrum antimicrobial potential. For instance, *Bacillus siamensis*, *B. velezensis*, and *B. subtilis* have been reported to suppress various fungal pathogens, and *B. subtilis* can also promote the growth of cut chrysanthemums by enhancing inter-microbial interactions (Sarfaraz et al., 2024; Sun et al., 2024; Tae An et al., 2024; Chandra et al., 2024; Wang M. et al., 2024; Tao et al., 2025). To explore the link between this microbial shift and soil biochemical functions, we performed a Spearman correlation analysis between the top 20 bacterial genera and soil enzyme activities (Supplementary Figure S1). The relative abundance of *Bacillus* was significantly and positively correlated with soil catalase (CAT) activity (*p* < 0.05), and this genus is a well-documented producer of extracellular enzymes, including catalases that mitigate oxidative stress (Zhang et al., 2025; He et al., 2025). Multiple other dominant taxa also exhibited positive correlations with CAT activity, suggesting a synergistic, community-level functional response to crop rotation. Nevertheless, we propose that *Bacillus*, enriched as a specific biomarker in the maize rotation and known for its enzymatic capacity, likely participates in and contributes to the enhanced CAT activity, reflecting a positive link between microbial community restructuring and improved soil enzymatic function.

Fungal biomarkers identified in the third crop of the continuous cropping group included *Paecilomyces* and *Curvularia geniculata.* Notably, certain species of *Paecilomyces* are known pathogens causing amaranth wilt and rose rot (Jin et al., 2025; Lahuf et al., 2022). *Curvularia geniculata* has been reported to induce tea leaf blight, rice panicle rot, as well as leaf spot diseases in coffee, *Bletilla striata*, and bananas (Biswajit et al., 2025; Zhu et al., 2025; Hyo et al., 2023; Xie et al., 2023; Qi et al., 2021). More critically, the relative abundance of the pathogenic genus *Fusarium* was highest in the continuous cropping group. The two rotation regimes effectively reduced this abundance by 28.1 and 17.1%, respectively. This finding aligns with the report by Ding, who observed during the continuous cropping of cut chrysanthemums, the total number of fungi decreased, while the abundance of *Fusarium* increased (Ding et al., 2023). As a well-established pathogen causing chrysanthemum wilt (Liu et al., 2023; Li W. et al., 2024; Dune et al., 2024) subsequent plantings. In contrast, neither the maize nor the cabbage rotation group exhibited enrichment of known major fungal pathogens, further supporting the beneficial role of rotation in suppressing harmful fungi. It should be noted that these reductions represent changes in relative abundance based on sequencing data. Future studies incorporating quantitative PCR (qPCR) are warranted to quantify the absolute abundance and total pathogen load. Additionally, while our sample size of three replicates per treatment limits statistical power, our composite sampling and RCBD design minimized pseudoreplication, and future studies with larger sample sizes are needed to validate our findings.

### Correlation between microorganisms and environmental factors

Research on soil microorganisms has greatly advanced our understanding of their interactions with the soil environment. Microbial processes critically influence the availability of soil nutrients, which in turn affects plant growth, while these nutrients also sustain the microbial community. Our study demonstrates that crop rotation can promote an increase in microbial richness, and the microbial community structure is more responsive to environmental changes than microbial diversity—a result consistent with the findings of Zhou et al. (2020).

Correlation analysis of the top 20 environmental factors revealed divergent responses between bacteria and fungi toward soil properties and plant phenotypes. Specifically, the correlation between bacterial communities and environmental factors was more universal, while the correlation of fungal communities was more specific. Notably, genera such as *Crustoderma*, *Plectania*, and *Cylindrocarpon* showed a significant negative correlation with plant phenotypic indicators (e.g., plant height) and other factors. These fungi have been rarely studied in agricultural contexts and may be potential factors that inhibit plant growth.

From the perspective of enzyme activity, catalase activity was the most influential factor on bacteria, while alkaline phosphatase activity had the greatest impact on fungi. However, the combined effect of multiple factors on microorganisms was limited for both groups, potentially due to interacting or counteracting effects among different enzymes. According to the RDA analysis, we concluded that nitrogen, phosphorus, and carbon are important factors affecting bacteria—this is consistent with the research results of Du et al. (2025). In contrast, fungal communities were predominantly influenced by phosphorus, calcium, and magnesium, and the role of trace elements (calcium and magnesium) should not be ignored either.

Mantel test results analysis showed that the external environment had a greater impact on bacteria; whereas fungal communities exhibited greater specificity, showing significant correlations only with specific variables such as pH and hydrolyzable nitrogen. This may be attributed to the multicellular filamentous structure of fungi, which can enhance their resilience to short-term environmental fluctuations compared to bacteria.

Phosphorus is an essential macronutrient for plant growth and development (Hu et al., 2025). It is involved in photosynthesis, respiration, and the formation of nucleic acids and cell membranes, and also serves as a critical substance for the functioning of various enzymes (Lambers, 2021). Moreover, studies by Cao have shown that phosphorus availability is a key factor in controlling *Fusarium* wilt (Cao et al., 2024). In our study, available phosphorus was significantly positively correlated with the plant height, stem diameter, and fresh stem weight of cut chrysanthemums. Meanwhile, it was also found that phosphorus is an important element affecting bacteria and fungi in the soil, which is consistent with the research results of Wu et al. (2022). Notably, the cabbage rotation group exhibited the highest soil available phosphorus content and, concurrently, no pathogenic fungal biomarkers were detected in this treatment. This combination of evidence suggests that cabbage rotation is a particularly effective cropping system for enhancing phosphorus availability and suppressing soil-borne fungal pathogens.

## Conclusion

This study demonstrates that crop rotation with maize or cabbage is a highly effective ecological strategy to mitigate continuous cropping obstacles in cut chrysanthemum. The primary benefits manifest in the comprehensive improvement of soil physicochemical properties, enhancement of enzyme activities, and optimization of the rhizosphere microbiota. While rotation significantly accelerated early vegetative growth, most differences in final plant biomass and key marketable traits (e.g., stem diameter and plant height) narrowed by harvest and were not statistically significant, with the sole exception of shoot fresh weight in the cabbage rotation group (Figure 1d). Therefore, the direct agronomic benefits of rotation are most pronounced during the early developmental stages. Crucially, both rotation systems successfully reduced the relative abundance of the primary pathogen *Fusarium*. Between the two, cabbage rotation was highly effective in accumulating soil phosphorus and broadly suppressing soil-borne pathogenic fungi. Maize rotation, on the other hand, excelled in regulating soil enzyme activities and enriching beneficial bacteria such as *Bacillus*. From an economic perspective, rotation maximizes land-use efficiency and mitigates the economic risks associated with monoculture. The additional harvest from rotation crops, combined with long-term savings in soil-borne disease management, effectively offsets the costs of rotation. Ultimately, this approach serves as a sustainable, soil health-centered strategy that balances soil ecological preservation with practical economic viability for the cut chrysanthemum industry.

## Data Availability

The high-throughput sequencing data related to this study can be accessed through NCBI with the accession number PRJNA1468310. All other data are included in this article and its additional files. for further inquiries, please contact the corresponding author.
