# Correction: Crop rotation patterns affect the growth, soil properties, and rhizosphere microbiome of cut chrysanthemums

**DOI:** 10.3389/fmicb.2026.1900191

**Published:** 2026-06-11

**Authors:** Frontiers Production Office

**Affiliations:** Frontiers Media SA, Lausanne, Switzerland

**Keywords:** chrysanthemum, continuous cropping obstacles, crop rotation, rhizosphere microorganisms, soil properties

The **Funding** statement was incorrect as published and did not include the grant number.

The incorrect **Funding** statement is below:

“The author(s) declared that financial support was not received for this work and/or its publication.”

The corrected **Funding** statement is below:

“The author(s) declared that financial support was received for this work and/or its publication. This work was supported by the Key Research and Development Program of Ningxia Hui Autonomous Region (2026BBF02015), and Key Laboratory of Biology and Genetic Improvement of Flower Crops (North China), Ministry of Agriculture and Rural Affairs.”

The **Author contribution** section did not include all appropriate contributions as published.

The incorrect **Author contribution** statement is below:

“CT: Writing – original draft. HL: Writing – review & editing. XS: Writing – review & editing. HG: Writing – review & editing. YK: Writing – original draft. SY: Writing – review & editing. RJ: Writing – review & editing. XZ: Writing – review & editing.”

The correct **Author contribution** statement is below:

“CT: Data curation, Investigation, Methodology, Project administration, Writing – review & editing, Formal analysis. HL: Conceptualization, Formal analysis, Writing – review & editing. XS: Writing – review & editing. HG: Conceptualization, Writing – review & editing, Methodology. YK: Writing – original draft, Conceptualization, Methodology. SY: Writing – review & editing, Conceptualization, Methodology, Project administration, Supervision. RJ: Writing – review & editing, Conceptualization, Methodology, Resources, Supervision. XZ: Supervision, Data curation, Writing – review & editing.”

The **Data availability statement** was incorrect as published.

The incorrect **Data availability statement** is below:

“Sequencing raw data is available on the NCBI, with the accession number PRJNA1468310.”

The correct **Data availability statement** is below:

“The high-throughput sequencing data related to this study can be accessed through NCBI with the accession number PRJNA1468310. All other data are included in this article and its additional files. for further inquiries, please contact the corresponding author.”

In the published article, Figures 1 and 2 were not published with the corrected image provided.

The correct Figures 1 and 2 are below:

**Figure 1:**
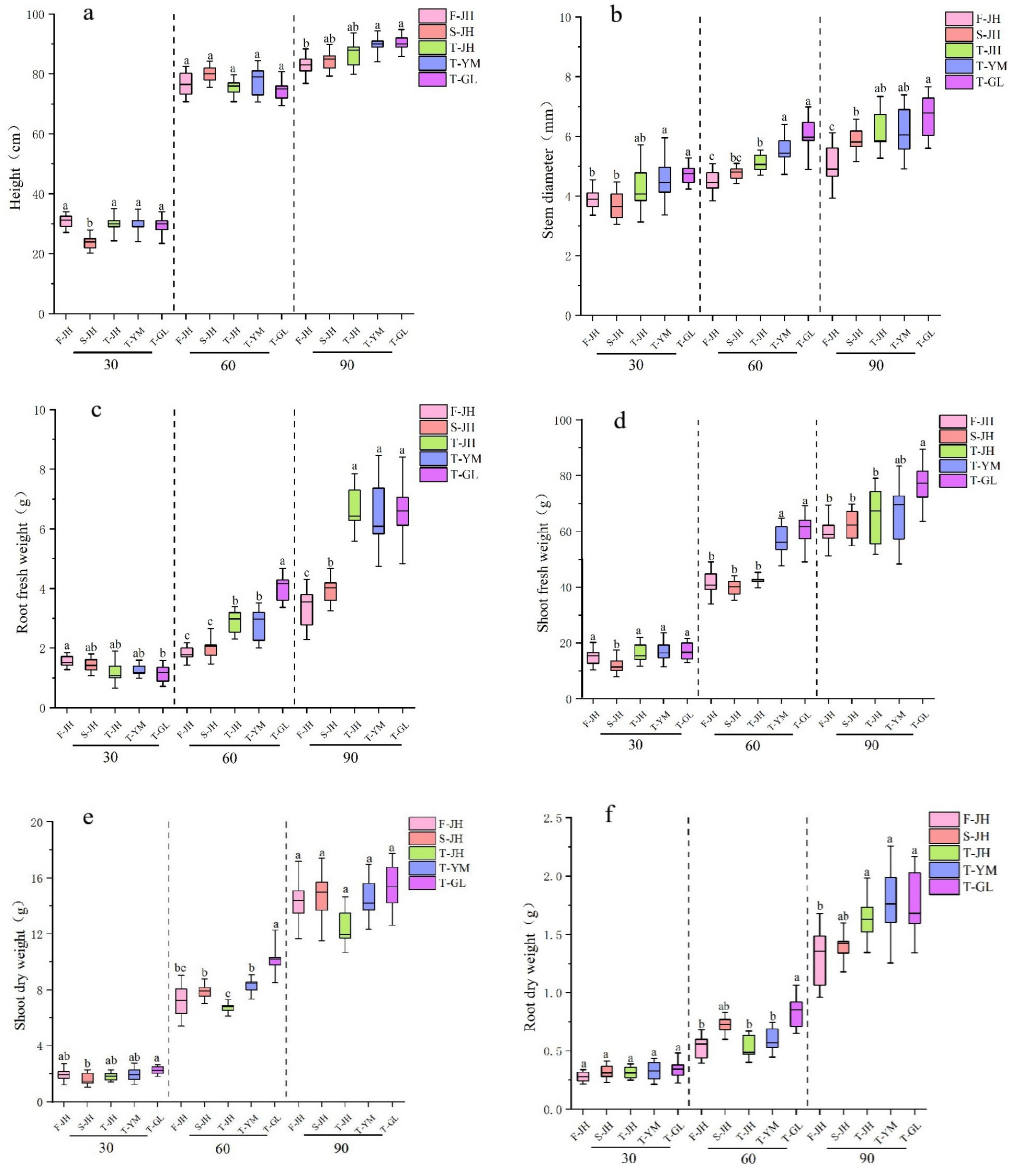
Phenotypic traits of cut chrysanthemum under different treatments: **(a)** Plant height; **(b)** stem diameter; **(c)** fresh root weight; **(d)** fresh stem weight; **(e)** dry stem weight; **(f)** dry root weight. F-JH, First crop of chrysanthemum group; S-JH, Second crop of chrysanthemum group; T-JH, Third crop of chrysanthemum group; T-YM, Third crop following maize rotation; T-GL, Third crop following cabbage rotation. Numbers 30, 60, and 90 represent the number of planting days. Different lowercase letters indicate significant differences (*p* < 0.05), and error bars represent standard deviations.

**Figure 2:**
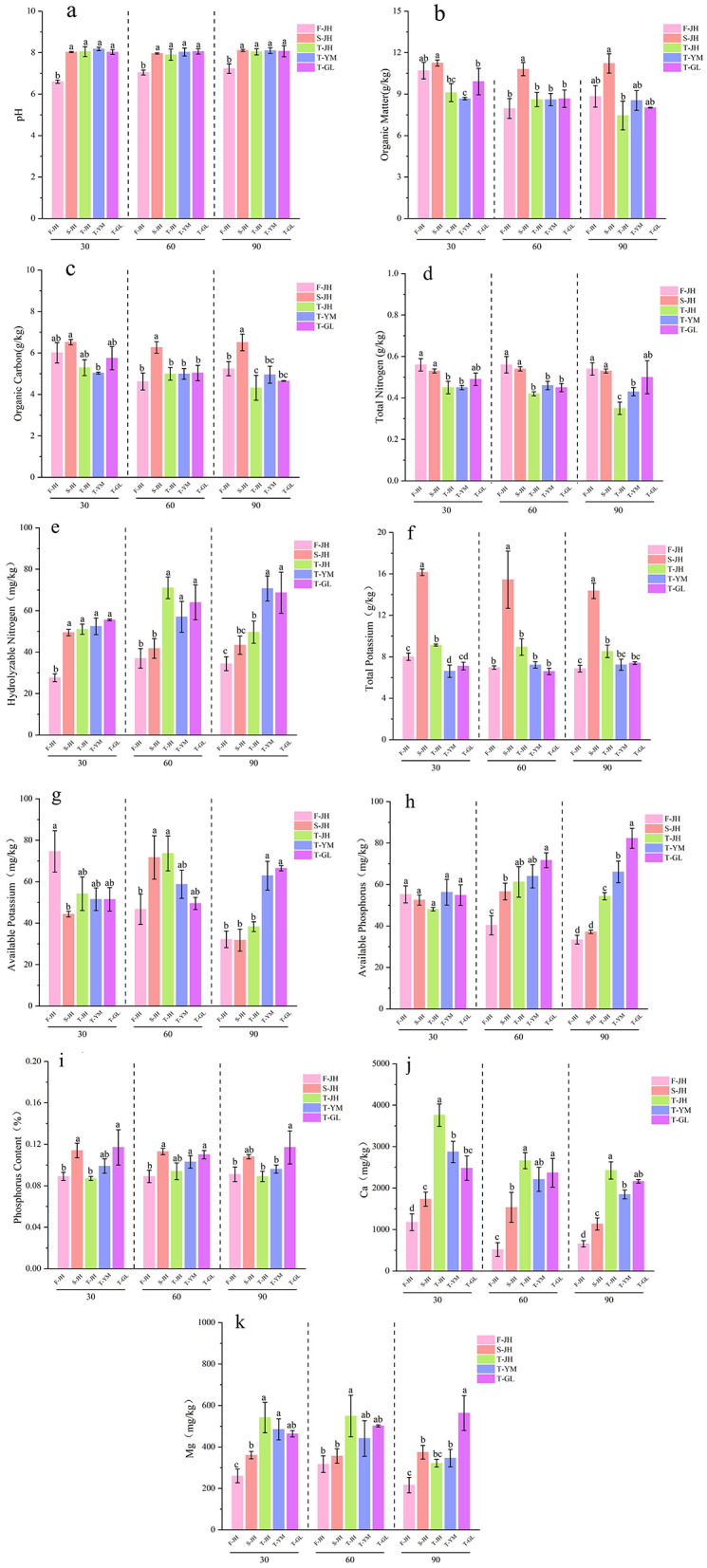
Soil physicochemical properties under different treatments: **(a)** pH; **(b)** Organic matter; **(c)** Organic carbon; **(d)** Total nitrogen; **(e)** Hydrolyzable nitrogen; **(f)** Total potassium; **(g)** Available potassium; **(h)** Available phosphorus; **(i)** Phosphorus content; **(j)** Calcium (Ca); **(k)** Magnesium (Mg). F-JH, First crop of chrysanthemum group; S-JH, Second crop of chrysanthemum group; T-JH, Third crop of chrysanthemum group; T-YM, Third crop following maize rotation; T-GL, Third crop following cabbage rotation. Here, 30, 60, and 90 represent the number of planting days. Different lowercase letters indicate significant differences (*p* < 0.05), and error bars represent standard deviations.

Numerous references did not include correct page ranges. These references have been updated accordingly.

A correction has been made to the **Results** section of the Abstract, page 1.

The incorrect sentence is below:

“At 60 days of growth, cut chrysanthemums under crop rotation systems exhibited significant increases stem in diameter, as well as fresh and dry weights of both aboveground and underground biomass, compared to continuous cropping.”

The correct sentence is below:

“At 60 days of growth, cut chrysanthemums under crop rotation systems exhibited significant increases in stem diameter, as well as fresh and dry weights of both aboveground and underground biomass, compared to continuous cropping.”

A correction has been made to the **Conclusion** section of the Abstract, page 1.

The incorrect sentence is below:

“Maize rotation excels in regulating soil enzyme activities and bacterial communities, whereas cabbage rotation is more effective in promoting nutritional growth period plant biomass, accumulating soil phosphorus, and inhibiting pathogenic fungi.”

The corrected sentence is below:

“Maize rotation excels in regulating soil enzyme activities and bacterial communities, whereas cabbage rotation is more effective in promoting plant biomass during the vegetative growth stage, accumulating soil phosphorus, and inhibiting pathogenic fungi.”

The original version of this article has been updated.

