# Supplementary material for: Crop rotation patterns affect the growth, soil properties, and rhizosphere microbiome of cut chrysanthemums
Source: Front Microbiol. 2026 May 28;17:1763144. doi: 10.3389/fmicb.2026.1763144 (PMC13296538; doi:10.3389/fmicb.2026.1763144)
Supplement: Supplementary file 1 [file Data_Sheet_1.docx]

Supplementary Material

1. **Supplementary Figures and Tables**

**1.1 Supplementary Figures**


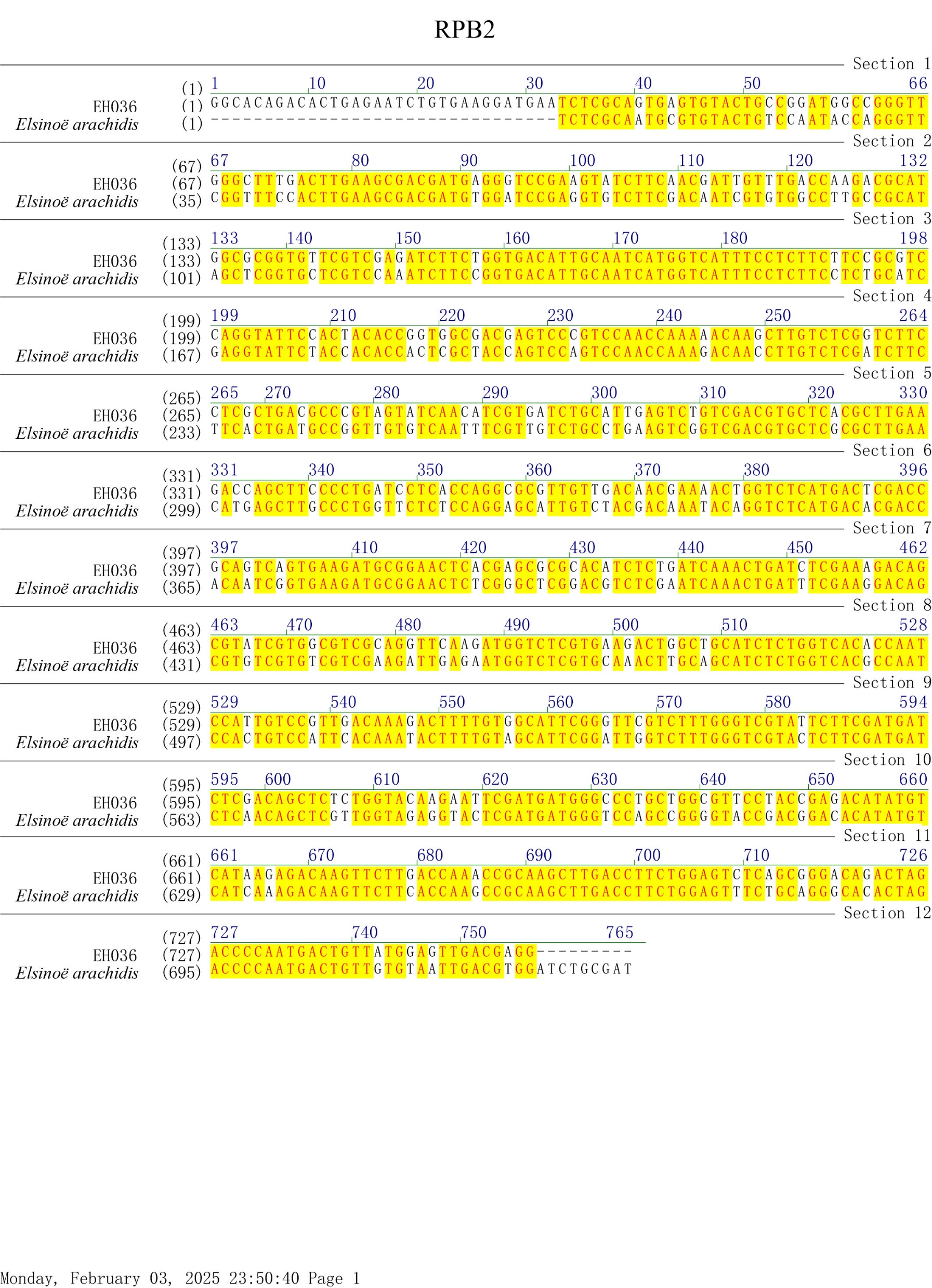

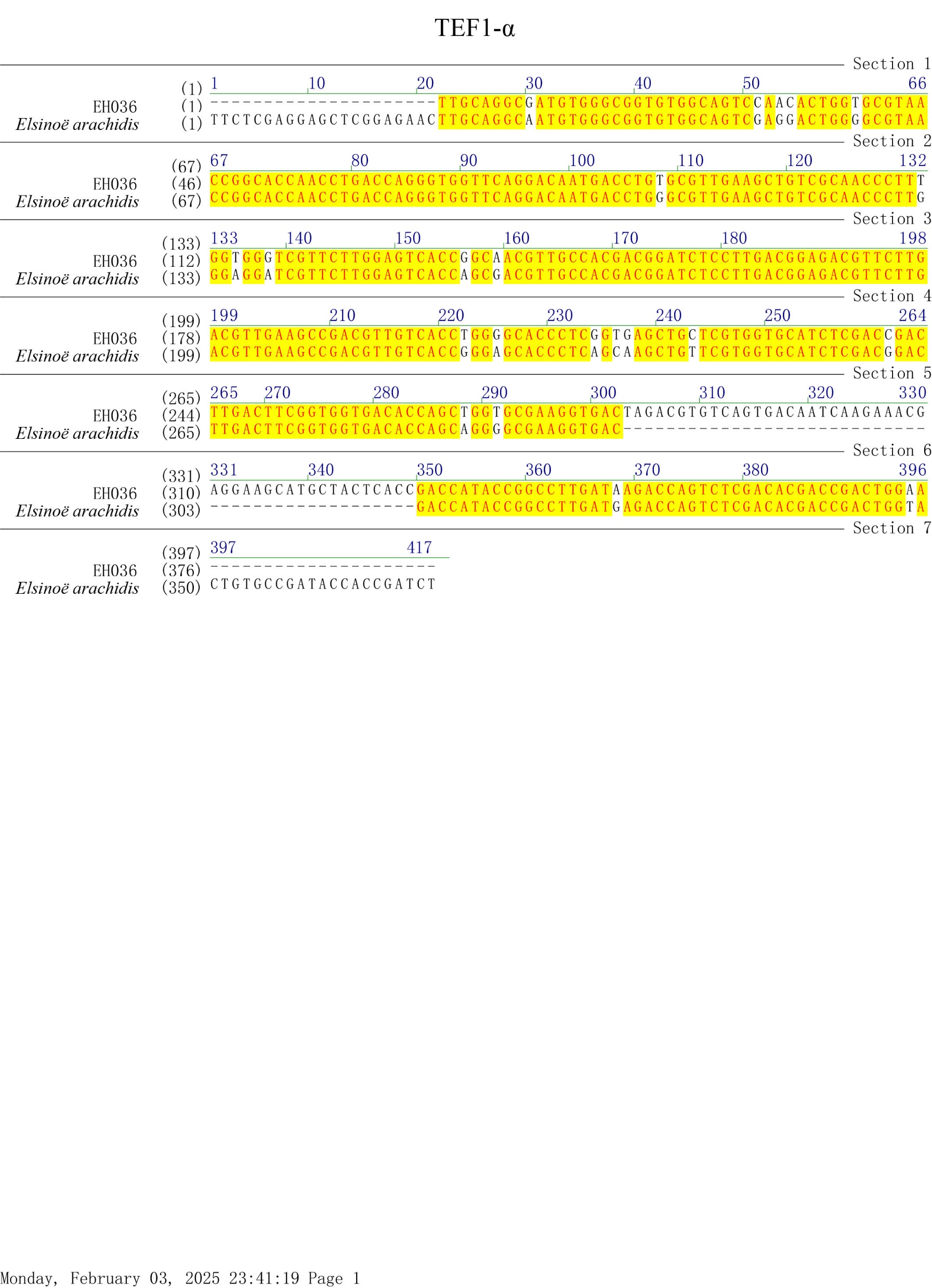

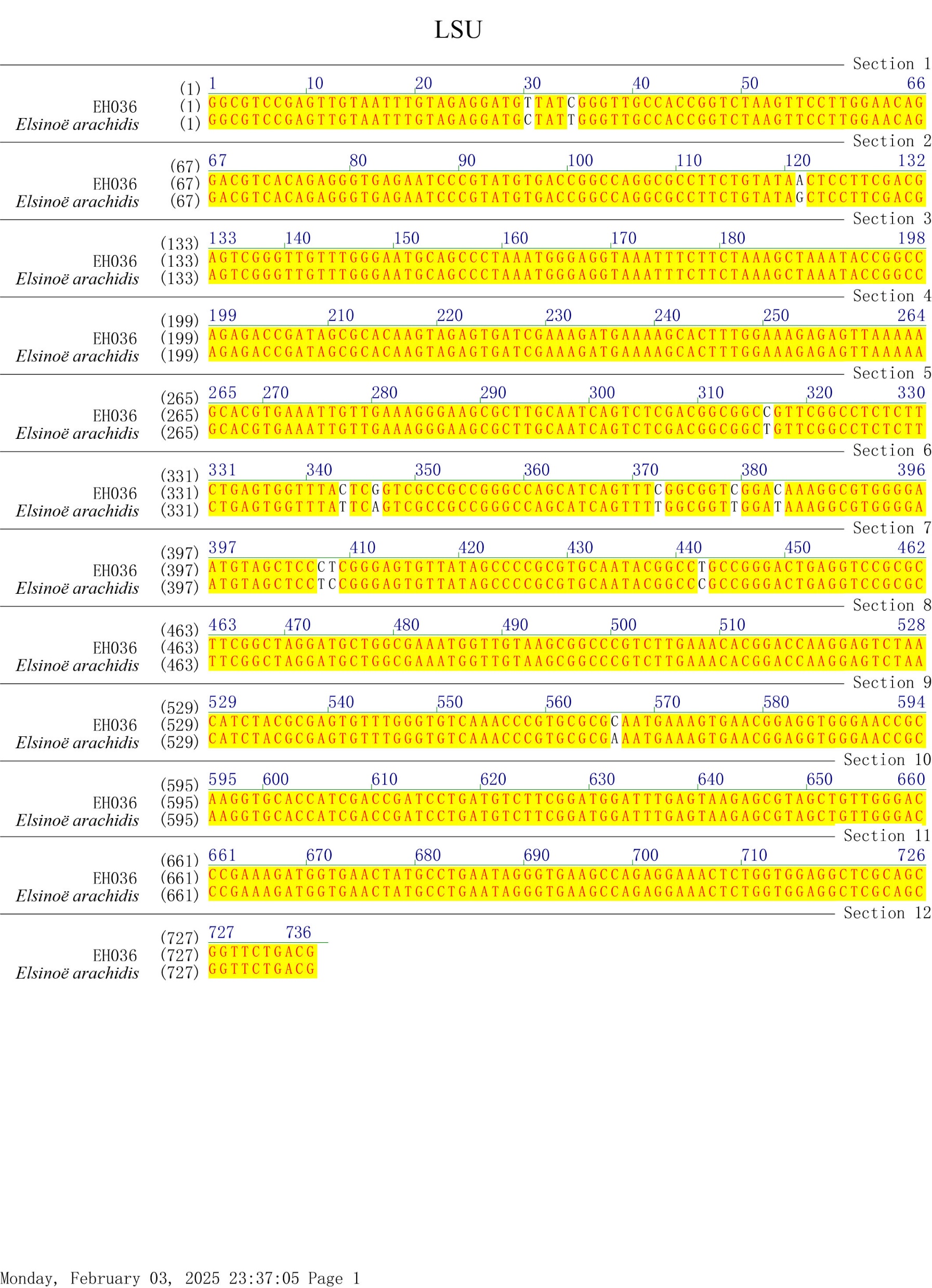

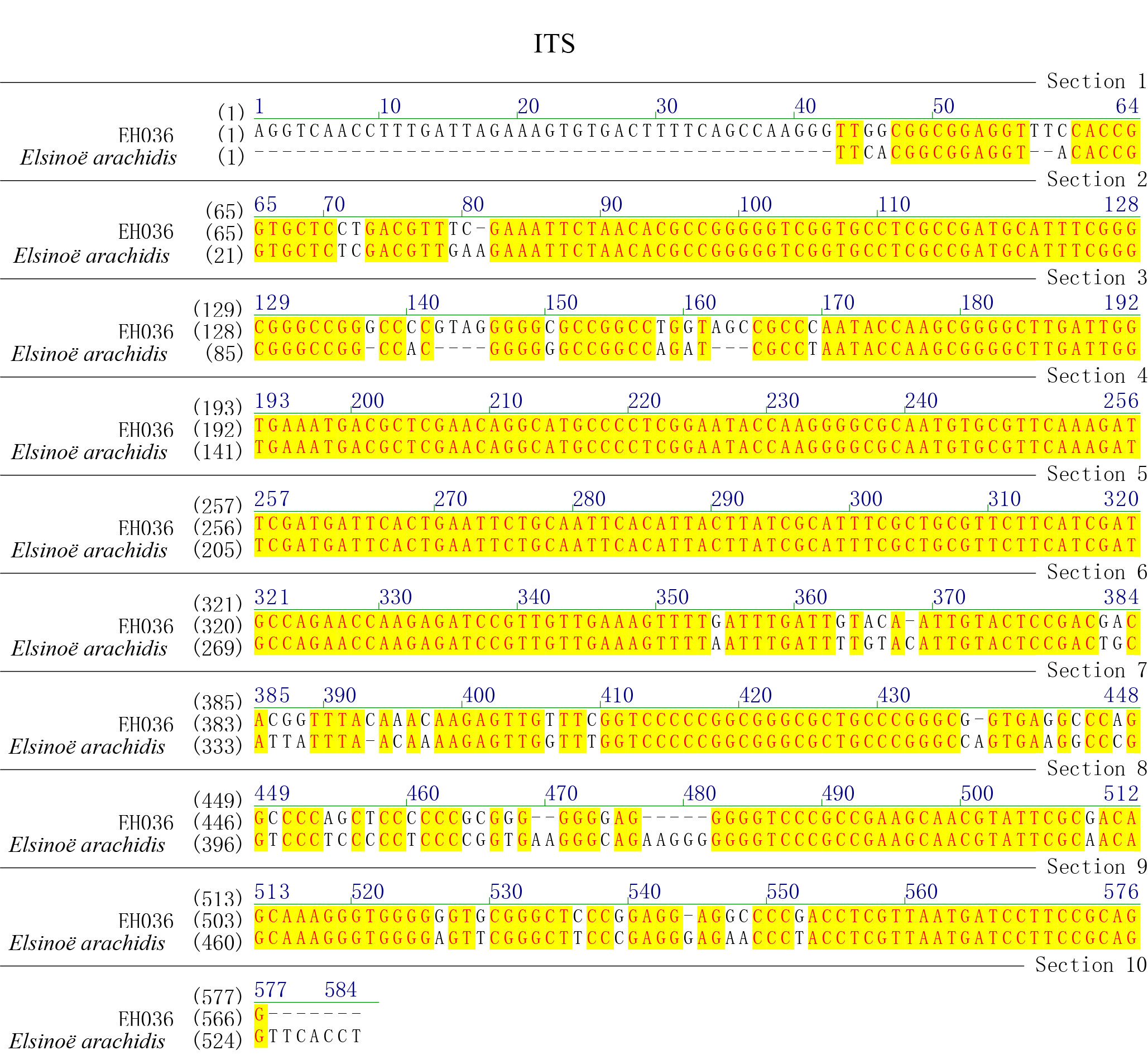


**A**

**B**

**C**

**D**

**Supplementary Figure S1** Alignments of ITS, LSU, *RPB2*, and *TEF1- α* sequences between EH036 and *Elsinoë arachidis*. A, ITS sequence alignment, 80.0% identity; B, LSU sequence alignment, 98.2% identity; C, *RPB2* sequence alignment, 76.9% identity. D, *TEF1- α* sequence alignment, 73.4% identity.


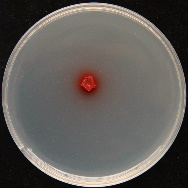

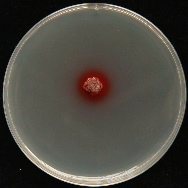

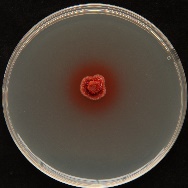

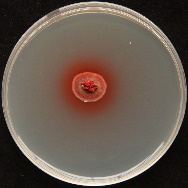

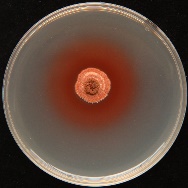

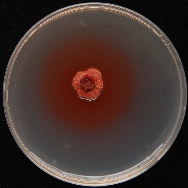

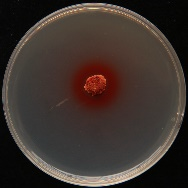

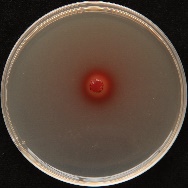


5°C


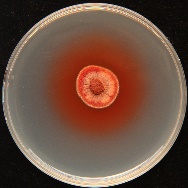


28°C

10°C

15°C

20°C

25°C

30°C

35°C

40°C


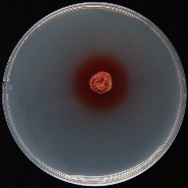

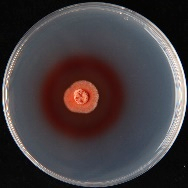

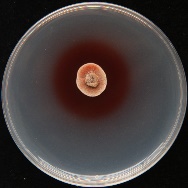

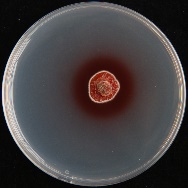

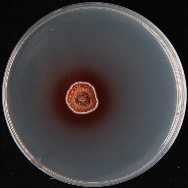

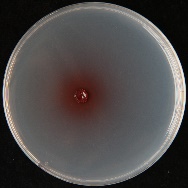

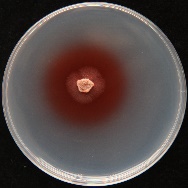


tryptone

yeast extract

(NH_4_)_2_SO_4_

leucine

sources without nitrogen

urea

casein acid hydrolysate


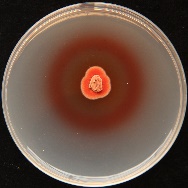

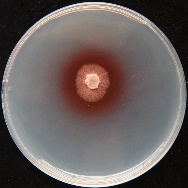

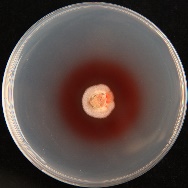

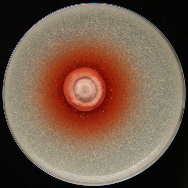

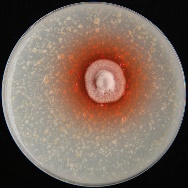

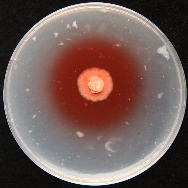

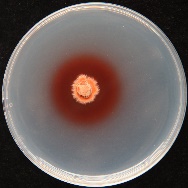

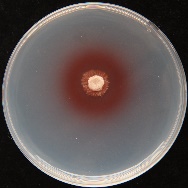


corn meal

glucose

lactose

maltose

sources without carbon

mannitol

soluble starch

oats


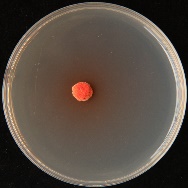

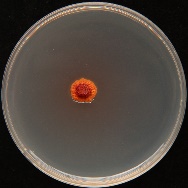

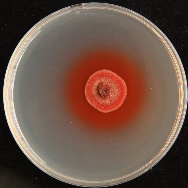

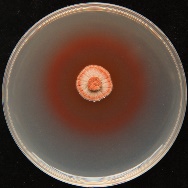

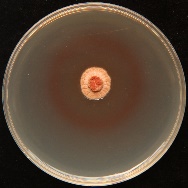

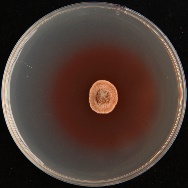

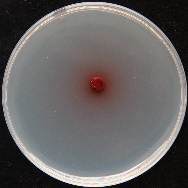


pH 2.0

pH 4.0

pH 7.0

pH 6.0

pH 8.0

pH 12.0

pH 10.0


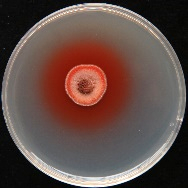

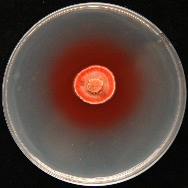

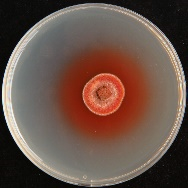


light

dark

light and dark

**Supplementary Figure S2** Optimization of cultivation conditions for isolate EH036.


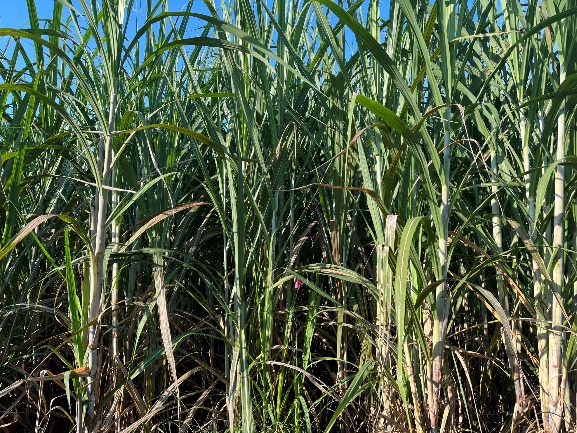

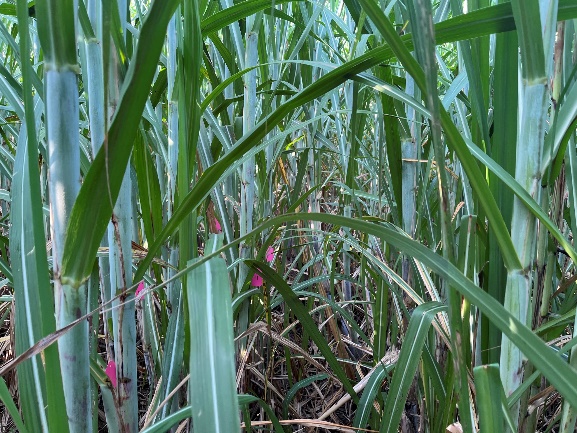

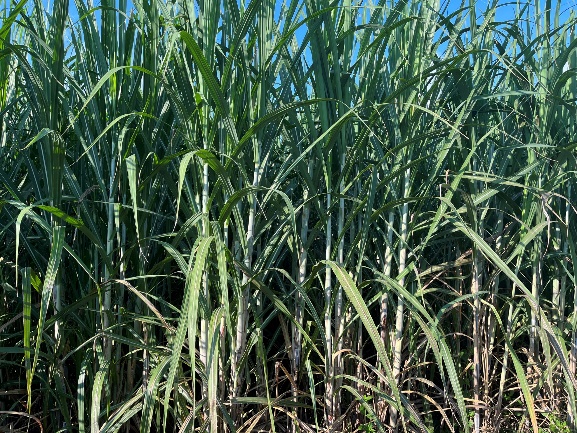

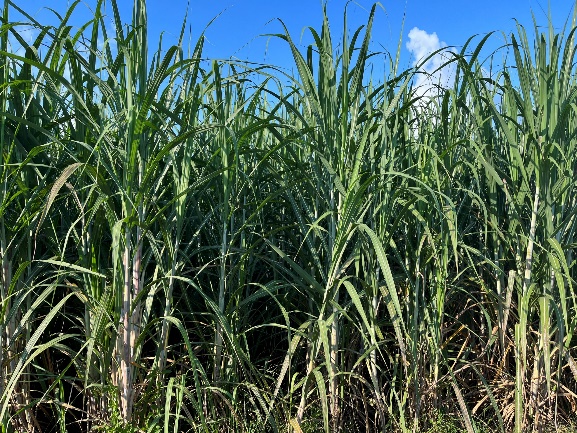

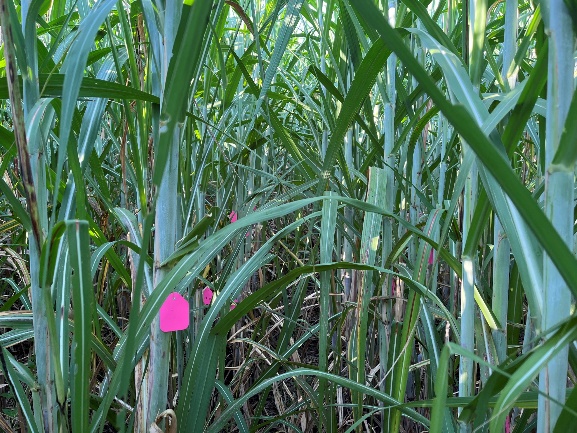

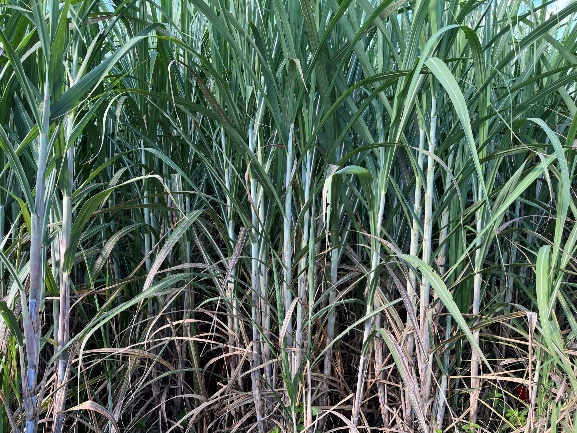

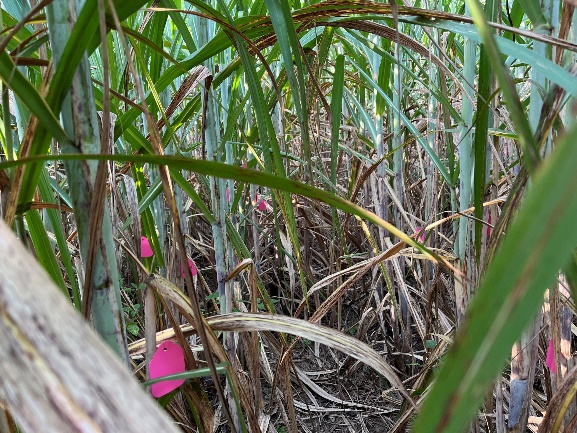

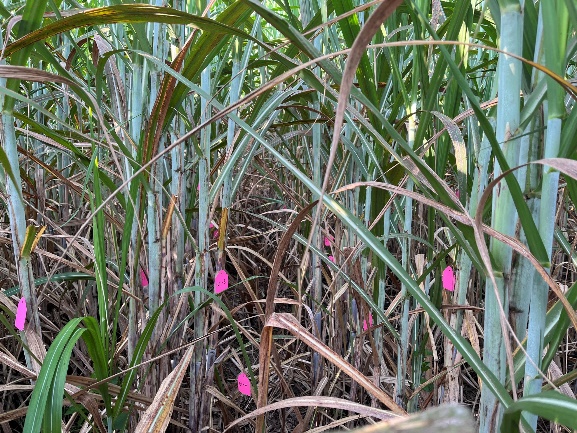

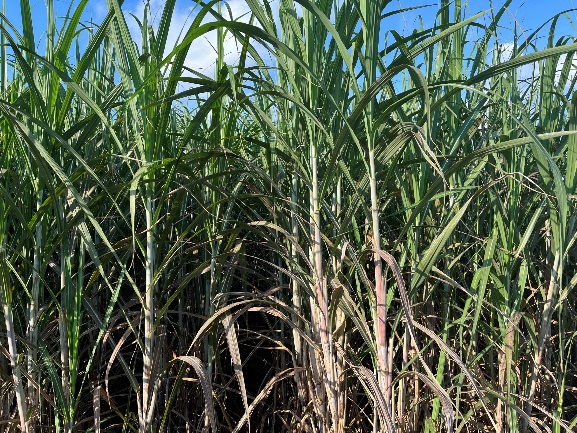


Myclobutanil

(0.0625 g/L)

Trifloxystrobin·tebuconazole (0.188 g/L)

CK

Thiophanate-methyl

(0.700 g/L)

Pyraclostrobin·boscalid (0.190 g/L)

Integral

Localized


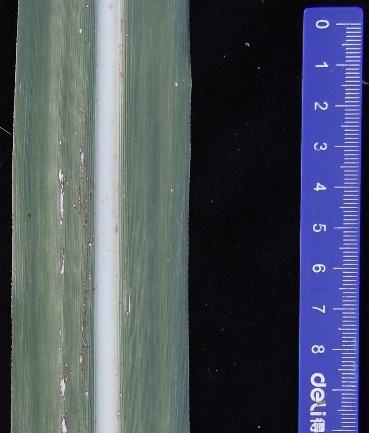

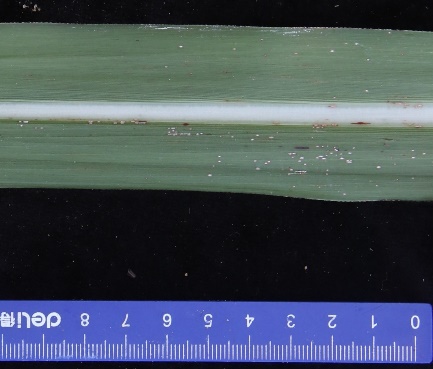

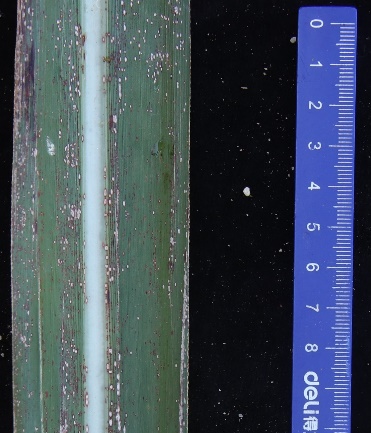

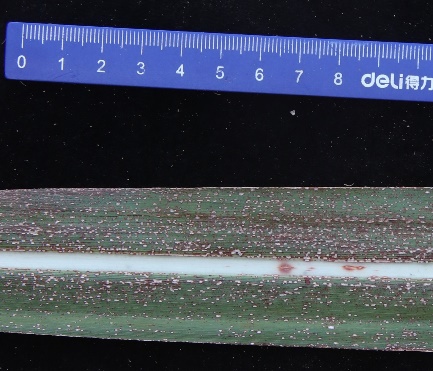


Single leaf


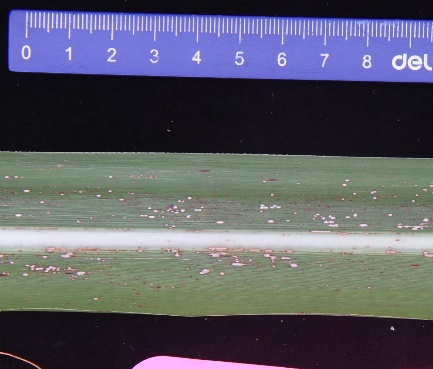

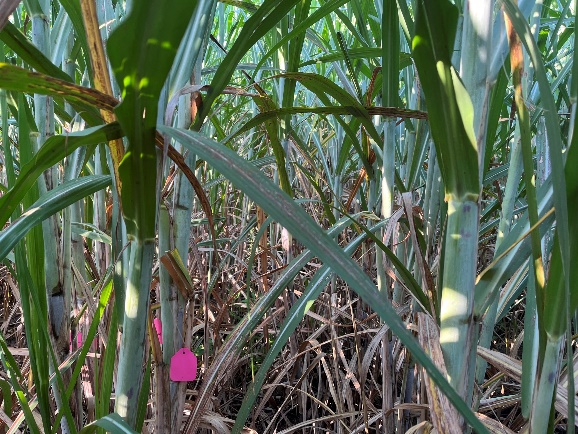


**Supplementary Figure S3.** Image of field control trail trials. Field investigation and photographic recording were performed at 10 days after the 3^rd^ spray of fungicide.

**1.2** **Supplementary Tables**

**Supplementary Table S1**. Fungicides and the concentration gradient used for EH036 mycelium growth rate and spore germination.

| **Fungicide name** | **Manufacture** | **Concentration Gradient (mg·L−1)** | |
| --- | --- | --- | --- |
|  |  | **inhibition of mycelial growth** | **inhibition of spore germination** |
| 20% Propiconazole ME | Hebei Zhongbao Green Crop Co., Ltd | 0.005, 0.01, 0.015, 0.02, 0.025 | 0.005, 0.025, 0.125, 0.625, 3.125 |
| 10% Difenoconazole ME | Hebei Zhongbao Green Crop Co., Ltd | 0.00125, 0.0025, 0.005, 0.01, 0.02 | 0.002, 0.01, 0.05, 0.25, 1.25 |
| 25% Myclobutanil EC | Hebei Zhongbao Green Crop Co., Ltd | 0.015, 0.03, 0.06, 0.12, 0.24 | 0.002, 0.01, 0.05, 0.25, 1.25 |
| 430g/L Tebuconazole SC | Bayer Crop Science (China) Co., Ltd | 0.008, 0.016, 0.032, 0.064, 0.128 | 0.02, 0.04, 0.08, 0.16, 0.32 |
| 3% Zhongshengmycin WP | Hebei Zhongbao Green Crop Co., Ltd | 0.027, 0.081, 0.243, 0.729, 2.187 | 0.025, 0.125, 0.625, 3.125, 6.25 |
| 25% Azoxystrobin SC | Hebei Zhongbao Green Crop Co., Ltd | 0.01, 0.05, 0.25, 1.25, 6.25 | 0.01, 0.05, 0.25, 1.25, 6.25 |
| 80% Mancozeb WP | Jinan Taihe Chemical Co., Ltd | 0.045, 0.135, 0.405, 1.215, 3.645 | 0.005, 0.015, 0.045, 0.135, 0.405 |
| 40% Pyrimethanil SC | Hebei Zhongbao Green Crop Co., Ltd | 0.09, 0.27, 0.81, 2.43, 7.29 | 0.09, 0.27, 0.81, 2.43, 7.29 |
| 50% Chlorobromoisocyanuric acid WP | Nanjing Nannong Pesticide Co., Ltd | 0.002, 0.006, 0.018, 0.054, 0.162 | 0.045, 0.135, 0.405, 1.215, 3.645 |
| 50% Carbendazim WP | Jiangsu Sanshan Pesticide Co., Ltd | 0.05, 0.075, 0.10, 0.125, 0.15 | 0.135, 0.405, 1.215, 3.645, 10.935 |
| 70% Thiophanate-methyl WP | Jinan Taihe Chemical Co., Ltd | 0.08, 0.16, 0.32, 0.64, 1.28 | 0.16, 0.32, 0.64, 1.28, 2.56 |
| 75% Chlorothalonil WP | Limin Chemical Co., Ltd | 0.09, 0.27, 0.81, 2.43, 7.29 | 0.01, 0.03 0.09, 0.27 0.81 |
| 75% Trifloxystrobin·tebuconazole WG | Bayer Crop Science (China) Co., Ltd | 0.008, 0.016, 0.032, 0.064, 0.128 | 0.0005, 0.001, 0.002, 0.004, 0.008 |
| 38% Pyraclostrobin·boscalid SC | Hebei Zhongbao Green Crop Co., Ltd | 0.002, 0.006, 0.018, 0.054, 0.162 | 0.0000625, 0.000125, 0.0005, 0.001 |
| 25% Pyraclostrobin SC | Hebei Zhongbao Green Crop Co., Ltd | 0.025, 0.05, 0.10, 0.20, 0.40 | 0.005, 0025, 0.125, 0.625, 3.125 |

**Supplementary Table S2.** Inhibition of mycelial growth in four *Elsinoë sacchari* isolates by fungicides

| **Strain name** | **Fungicides** | **Toxicity equation** | **R^2^** | **EC_50_ (mg/L)** | **95% Confidence Intervals (mg/L)** |
| --- | --- | --- | --- | --- | --- |
| EH007 | Pyraclostrobin·boscalid | y=6.60+0.87x | 0.980 | 0.0145 | 0.00949~0.0231 |
|  | Trifloxystrobin·tebuconazole | y=8.80+2.22x | 0.996 | 0.0194 | 0.0165~0.0223 |
|  | Myclobutanil | y=7.03+1.41x | 0.974 | 0.0363 | 0.0267~0.0438 |
|  | Thiophanate-methyl | y=6.81+3.10x | 0.983 | 0.261 | 0.200~0.320 |
| EH013 | Pyraclostrobin·boscalid | y=6.91+1.17x | 0.999 | 0.0233 | 0.022~0.0244 |
|  | Trifloxystrobin·tebuconazole | y=9.70+2.52x | 0.978 | 0.0136 | 0.0110~0.0162 |
|  | Myclobutanil | y=8.25+2.20x | 0.929 | 0.0333 | 0.0247~0.0440 |
|  | Thiophanate-methyl | y=7.00+3.30x | 0.977 | 0.248 | 0.165~0.310 |
| EH017 | Pyraclostrobin·boscalid | y=8.65+1.89x | 0.925 | 0.0117 | 0.00888~0.0273 |
|  | Trifloxystrobin·tebuconazole | y=9.19+2.47x | 0.99 | 0.0201 | 0.0149~0.0249 |
|  | Myclobutanil | y=6.84+1.42x | 0.991 | 0.0506 | 0.0411~0.0597 |
|  | Thiophanate-methyl | y=6.67+2.97x | 0.953 | 0.274 | 0.180~0.409 |
| EH032 | Pyraclostrobin·boscalid | y=7.93+1.47x | 0.923 | 0.0102 | 0.00479~0.0281 |
|  | Trifloxystrobin·tebuconazole | y=9.34+2.25x | 0.953 | 0.0118 | 0.00939~0.0124 |
|  | Myclobutanil | y=7.46+1.92x | 0.944 | 0.0523 | 0.0376~0.0844 |
|  | Thiophanate-methyl | y=6.61+2.74x | 0.969 | 0.259 | 0.187~0.353 |

R^2^ = correlation coefficient; EC_50_ = median effect concentration

**Supplementary Table S3**. Inhibition of spore germination in four *Elsinoë sacchari* isolates by fungicides

| **Strain name** | **Fungicides** | **Toxicity equation** | **R^2^** | **EC_50_ (mg/L)** | **95% Confidence Intervals (mg/L)** |
| --- | --- | --- | --- | --- | --- |
| EH007 | Pyraclostrobin·boscalid | y=11.51+1.80x | 0.989 | 0.000242 | 0.000212~0.000300 |
|  | Trifloxystrobin·tebuconazole | y=9.82+1.86x | 0.993 | 0.00256 | 0.00214~0.00288 |
|  | Myclobutanil | y=4.92+0.80x | 0.953 | 1.259 | 0.586~3.969 |
|  | Thiophanate-methyl | y=3.59+0.50x | 0.952 | 660.693 | 67.453~1745.822 |
| EH013 | Pyraclostrobin·boscalid | y=10.77+1.64x | 0.999 | 0.000303 | 0.000285~0.000316 |
|  | Trifloxystrobin·tebuconazole | y=10.13+1.94x | 0.996 | 0.00227 | 0.00194~0.00262 |
|  | Myclobutanil | y=10.77+1.64x | 0.999 | 0.000303 | 0.000285~0.000316 |
|  | Thiophanate-methyl | y=3.78+0.49x | 0.993 | 308.884 | 74.302~1059.254 |
| EH017 | Pyraclostrobin·boscalid | y=11.05+1.69x | 0.997 | 0.000263 | 0.000250~0.000285 |
|  | Trifloxystrobin·tebuconazole | y=9.67+1.79x | 0.993 | 0.00246 | 0.00201~0.00281 |
|  | Myclobutanil | y=4.87+0.76x | 0.97 | 1.483 | 0.806~3.425 |
|  | Thiophanate-methyl | y=3.69+0.45x | 0.992 | 814.913 | 118.850~1267.652 |
| EH032 | Pyraclostrobin·boscalid | y=10.6+1.65x | 0.996 | 0.000404 | 0.000356~0.000471 |
|  | Trifloxystrobin·tebuconazole | y=9.81+1.81x | 0.993 | 0.00220 | 0.00180~0.00250 |
|  | Myclobutanil | y=4.84+0.81x | 0.98 | 1.576 | 0.741~3.562 |
|  | Thiophanate-methyl | y=3.65+0.50x | 0.928 | 501.187 | 17.783~696.627 |

R^2^ = correlation coefficient; EC_50_ = median effect concentration
